# Cortical mean diffusivity detects early age-related changes and associates with cognition and plasma biomarkers

**DOI:** 10.1093/braincomms/fcaf511

**Published:** 2026-01-10

**Authors:** Oriol Perera-Cruz, Cristina Solé-Padullés, Lídia Mulet-Pons, María Cabello-Toscano, Rachel M Morse, Kilian Abellaneda-Pérez, Rubén Perellón-Alfonso, Gabriele Cattaneo, Javier Solana-Sánchez, Vanessa Alviarez-Schulze, Nuria Bargalló, Juan Fortea, Josep M Tormos, Alvaro Pascual-Leone, Henrik Zetterberg, Lídia Vaqué-Alcázar, David Bartrés-Faz

**Affiliations:** Department of Medicine, Faculty of Medicine and Health Sciences and Institute of Neurosciences, University of Barcelona, Barcelona 08036, Spain; August Pi I Sunyer Institute of Biomedical Research (IDIBAPS), Barcelona 08036, Spain; Department of Medicine, Faculty of Medicine and Health Sciences and Institute of Neurosciences, University of Barcelona, Barcelona 08036, Spain; August Pi I Sunyer Institute of Biomedical Research (IDIBAPS), Barcelona 08036, Spain; Department of Medicine, Faculty of Medicine and Health Sciences and Institute of Neurosciences, University of Barcelona, Barcelona 08036, Spain; August Pi I Sunyer Institute of Biomedical Research (IDIBAPS), Barcelona 08036, Spain; Department of Medicine, Faculty of Medicine and Health Sciences and Institute of Neurosciences, University of Barcelona, Barcelona 08036, Spain; August Pi I Sunyer Institute of Biomedical Research (IDIBAPS), Barcelona 08036, Spain; Department of Medicine, Faculty of Medicine and Health Sciences and Institute of Neurosciences, University of Barcelona, Barcelona 08036, Spain; August Pi I Sunyer Institute of Biomedical Research (IDIBAPS), Barcelona 08036, Spain; Guttmann Institute, Institut Universitari de Neurorehabilitació adscrit a la Universitat Autònoma de Barcelona, Badalona 08916, Spain; Department of Medicine, Universitat Autònoma de Barcelona, Bellaterra, Cerdanyola del Vallès 08193, Spain; Fundació Institut d’Investigació en Ciències de la Salut Germans Trias i Pujol, Badalona 08916, Spain; August Pi I Sunyer Institute of Biomedical Research (IDIBAPS), Barcelona 08036, Spain; Guttmann Institute, Institut Universitari de Neurorehabilitació adscrit a la Universitat Autònoma de Barcelona, Badalona 08916, Spain; Fundació Institut d’Investigació en Ciències de la Salut Germans Trias i Pujol, Badalona 08916, Spain; Unit for Cognitive Neuroscience, Institute of Neurosciences, University of Barcelona, Barcelona 08035, Spain; Centre for Biomedical Research on Mental Health (CIBERSAM), Instituto de Salud Carlos III, Madrid 28029, Spain; Guttmann Institute, Institut Universitari de Neurorehabilitació adscrit a la Universitat Autònoma de Barcelona, Badalona 08916, Spain; Fundació Institut d’Investigació en Ciències de la Salut Germans Trias i Pujol, Badalona 08916, Spain; Guttmann Institute, Institut Universitari de Neurorehabilitació adscrit a la Universitat Autònoma de Barcelona, Badalona 08916, Spain; Fundació Institut d’Investigació en Ciències de la Salut Germans Trias i Pujol, Badalona 08916, Spain; Guttmann Institute, Institut Universitari de Neurorehabilitació adscrit a la Universitat Autònoma de Barcelona, Badalona 08916, Spain; Fundació Institut d’Investigació en Ciències de la Salut Germans Trias i Pujol, Badalona 08916, Spain; Department of Medicine, Faculty of Medicine and Health Sciences and Institute of Neurosciences, University of Barcelona, Barcelona 08036, Spain; August Pi I Sunyer Institute of Biomedical Research (IDIBAPS), Barcelona 08036, Spain; Centre for Biomedical Research on Mental Health (CIBERSAM), Instituto de Salud Carlos III, Madrid 28029, Spain; Neuroradiology Section, Radiology Department, Diagnostic Image Center, Hospital Clinic of Barcelona, University of Barcelona, Barcelona 08036, Spain; Sant Pau Memory Unit, Department of Neurology, Institut d’Investigacions Biomèdiques Sant Pau-Hospital de Sant Pau, Barcelona 08025, Spain; Barcelona Down Medical Center, Fundació Catalana de Síndrome de Down, Barcelona 08029, Spain; Guttmann Institute, Institut Universitari de Neurorehabilitació adscrit a la Universitat Autònoma de Barcelona, Badalona 08916, Spain; Centro de Investigación Traslacional San Alberto Magno, Universidad Católica de Valencia San Vicente Mártir, València 46001, Spain; Hinda and Arthur Marcus Institute for Aging Research and Deanna and Sidney WolkCenter for Memory Health, Hebrew SeniorLife, Harvard Medical School, Boston, MA 02131, USA; Department of Neurology, Harvard Medical School, Boston, MA 02115, USA; Department of Psychiatry and Neurochemistry, Sahlgrenska Academy, Institute of Neuroscience and Physiology, University of Gothenburg, Gothenburg 41390, Sweden; Department of Neurodegenerative Disease, UCL Institute of Neurology, London LN WC1N3BG, UK; Clinical Neurochemistry Laboratory, Sahlgrenska University Hospital, Mölndal 43180, Sweden; Department of Pathology and Laboratory Medicine, University of Wisconsin School of Medicine and Public Health, Madison, WI 53792, USA; Wisconsin Alzheimer’s Disease Research Center, University of Wisconsin School of Medicine and Public Health, University of Wisconsin-Madison, Madison, WI 53792, USA; UK Dementia Research Institute at UCL, London W1B 3HH, UK; Hong Kong Center for Neurodegenerative Diseases, InnoHK, Tseung Kwan O, Hong Kong, China; Centre for Brain Research, Indian Institute of Science, Bangalore, Karnataka 560012, India; Department of Medicine, Faculty of Medicine and Health Sciences and Institute of Neurosciences, University of Barcelona, Barcelona 08036, Spain; August Pi I Sunyer Institute of Biomedical Research (IDIBAPS), Barcelona 08036, Spain; Sant Pau Memory Unit, Department of Neurology, Institut d’Investigacions Biomèdiques Sant Pau-Hospital de Sant Pau, Barcelona 08025, Spain; Department of Medicine, Faculty of Medicine and Health Sciences and Institute of Neurosciences, University of Barcelona, Barcelona 08036, Spain; August Pi I Sunyer Institute of Biomedical Research (IDIBAPS), Barcelona 08036, Spain; Guttmann Institute, Institut Universitari de Neurorehabilitació adscrit a la Universitat Autònoma de Barcelona, Badalona 08916, Spain

**Keywords:** cortical mean diffusivity, cortical thickness, plasma biomarkers, cognition, magnetic resonance imaging

## Abstract

The relationship between age-related cognitive changes and cortical macrostructural properties [i.e. cortical thickness (CTh)] has been extensively studied. However, less is known about the relationship with cortical microstructural characteristics [i.e. cortical mean diffusivity (cMD)] even though these are sensitive to preclinical phases of Alzheimer’s disease. We studied a sample of 964 cognitively healthy adults (age: 40–82 years; 52% females) with available structural and diffusion MRI data. The preclinical Alzheimer’s cognitive composite was used as the cognition measure, and plasma concentrations of neurodegenerative-related (i.e. phosphorylated tau 181 and neurofilament light) and inflammatory (i.e. high-sensitivity C-reactive protein) biomarkers were assessed, together with apolipoprotein ɛ4 status. Neuroimaging data was preprocessed using FreeSurfer and FSL, and a homemade surface-based approach was used to obtain cMD maps. A two-class generalized linear model was used as the main statistical analysis. We identified a significant negative association between both cortical measures (cMD and CTh) and age. cMD associations were more extensive at earlier ages (<50 years), while CTh associations were greater at older ages (>50 years). cMD was positively correlated with cognition and with both neurodegenerative-related biomarkers in prefrontal regions, while the association was negative and more widespread for the inflammatory biomarker. CTh was positively correlated with cognition in more restricted areas than cMD and only negatively correlated with neurofilament light. Also, cMD presented lower levels in apolipoprotein ɛ4 carriers compared to non-carriers, while no results were found for CTh. Correlating cMD with CTh resulted in a regional pattern of negative and positive correlations, differencing somatosensory and associative areas, respectively. Altogether, we show that cMD can capture microstructural cortical changes occurring across adulthood into older age before CTh alterations. Indeed, it seems more sensitive to age-related cognitive decline and pathological and inflammatory processes related to risk profiles, showing an opposite trend to CTh in relation to neurodegenerative biomarker levels. Furthermore, our results suggest a pattern relating the two cortical metrics perhaps reflecting a cortical organization pattern.

## Teaser

Perera-Cruz *et al*. report how cortical mean diffusivity, a metric representative of microstructural characteristics, cross-sectionally decreases along the adult lifespan in 964 cognitively healthy individuals, presenting higher sensitivity to cognition measures, plasma neurodegenerative- and inflammation-related biomarkers and *APOE ɛ4* status than cortical thickness and also possibly identifying cortical organization patterns.

## Graphical Abstract

**Graphical Abstract:**
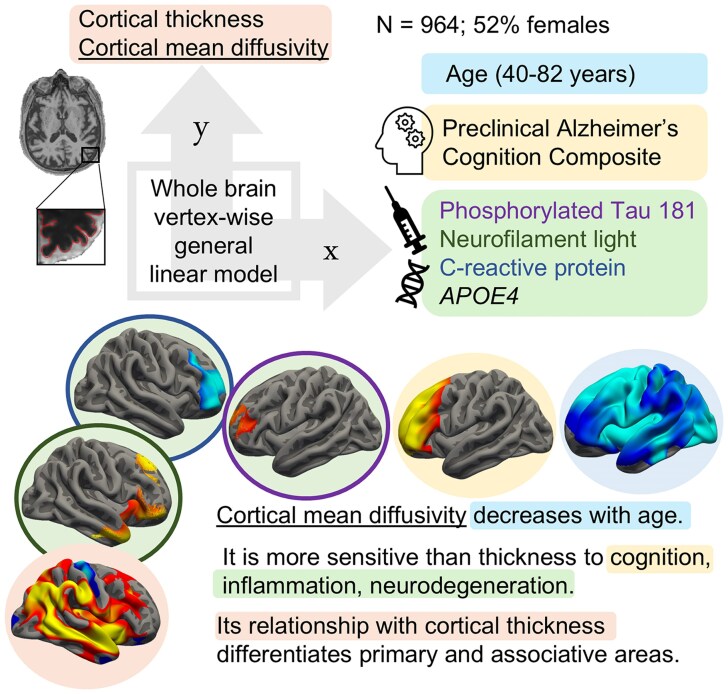

## Introduction

The demographic changes seen in recent years due to an increased life expectancy pose a challenge to health systems around the world, since advanced age is the main risk factor for a number of diseases, such as Alzheimer’s disease,^1,2^ and is associated with a naturally occurring cognitive change,^3-5^ where different individual patterns across time have been described, showing trajectories with declining or maintaining directions and high interindividual differences.^6,7^ These cognitive changes are linked to structural alterations in the brain that are measurable through brain imaging techniques such as MRI. In this context, midlife (40–65), where health problems start to emerge,^8^ is a crucial period of study to discern patterns of early change.^9,10^ Mainly, structural age-related changes in brain integrity have been studied through metrics such as grey matter (GM) volume or cortical thickness (CTh), evidencing progressive atrophy in older age.^11-13^ On the other hand, diffusion metrics characterizing the microstructure of the cortex have been less investigated in this context, despite their potential to identify earlier alterations prior to volumetric loss.^14^ Mean diffusivity (MD) is a diffusion tensor imaging (DTI)-derived metric that assesses the movement of water molecules in any direction within the three-dimensional space. When applied to the cortex [i.e. cortical mean diffusivity (cMD)], it is indicative of microstructural changes such as inflammation or atrophy, and it could prove more sensitive than CTh to age-related changes and initial pathology-related alterations.^15-17^ cMD could be able to detect a cortical inflammatory response related to the building-up of low-grade inflammation levels occurring in ageing, leading to a concomitant proinflammatory state, as posed in the ‘inflammaging’ hypothesis.^18,19^ Indeed, previous studies have seen lower values in diffusion metrics in GM in presymptomatic Alzheimer’s disease patients,^20,21^ suggesting an early inflammatory phase, although other studies have failed to detect it.^22^ However, fewer data is available regarding cognitively unimpaired elders,^23-25^ highlighting the need to investigate diffusion changes across broader age ranges and earlier life stages in healthy populations. Beyond identifying preclinical cases, discerning cognitive change patterns in midlife is paramount to understand the biological basis of brain health maintenance and to identify brain processes directly related to cognitive changes manifested later in older age.^26^ Interventions in those periods could not only contribute to reducing dementia incidence but also perhaps revert declining cognitive trajectories.

During recent years, interest in plasma biomarkers has increased as they are less invasive, more cost-effective and of wider acceptability and applicability and could be more sensitive for early Alzheimer’s disease diagnosis than CSF markers.^27-29^ In this context, plasma tau phosphorylated at threonine position 181 (pTau181) has been proposed as a specific biomarker for Alzheimer’s disease, since its levels rise early and gradually following the disease’s progression, and relate to amyloid beta (Aβ) and tau accumulation in the brain^23,30-33^ in diverse populations.^34^ Another widely used plasma biomarker is neurofilament light (NfL), a non-specific marker of neuronal degeneration and axonal damage,^35^ which has been shown to positively correlate with structural^29^ and cognitive^31,32,35^ changes along the Alzheimer’s disease continuum. Additionally, the inflammatory biomarker C-reactive protein (CRP) seems to increase across the adult lifespan^36,37^ and has been associated with cognitive impairment^38-41^ and the progression to dementia in the general population.^42,43^ Nonetheless, although a body of literature relates peripheral CRP with white matter (WM),^44,45^ the relationship between inflammatory biomarkers and cMD has been little explored, especially in ageing research. Another important determinant of cognitive health in midlife and older age is the presence of the apolipoprotein ɛ4 (*APOE ɛ4*) allele, the single most relevant genetic risk factor for Alzheimer’s disease.^46,47^ Carrier individuals have been shown to present faster cognitive decline^48^ in a biomarker-influenced manner,^49^ and altered GM diffusion characteristics,^50-52^ pointing to differential inflammatory responses and neurodegenerative processes worth exploring further.

Overall, characterizing how cMD and CTh vary jointly in a cognitively unimpaired sample from adulthood (40–65) until old age (over 65), and how they relate to cognition, plasma biomarkers, risk profiles, and to each other might be informative of brain changes and processes related to brain health maintenance and the variability observed in cognitive trajectories along the lifespan.^26,53^ Thus, our aims were (i) to characterize how cMD varies across the adult and older lifespan in a cognitively unimpaired sample and whether cMD could identify earlier changes than CTh; (ii) to assess whether cMD and CTh are associated with cognitive performance [i.e. preclinical Alzheimer’s cognitive composite (PACC) score], pathology-related plasma biomarkers (i.e. pTau181 and NfL), inflammation levels [i.e. high-sensitivity CRP (hsCRP)] and *APOE* status; and (iii) to investigate how cMD is associated with CTh and whether the relationship between the two integrity metrics is influenced by the levels of pathology-related or inflammation biomarkers. We expected cMD to be more sensitive than CTh in detecting subtle age-related cortical changes and overall cortical integrity to decline with age, which we predicted would also be associated with lower cognitive performance. We hypothesized higher cMD and lower CTh would be associated with elevated levels of pathology-related plasma biomarkers (pTau181 and NfL), whereas lower cMD and higher CTh would be associated with elevated levels of inflammation-related markers (hsCRP), which might be more evident in risk profile comparisons (*APOΕ ɛ4* carriers). We also anticipated that CTh and cMD would show distinct associations between them, influenced by both age and biomarker levels, considering atrophic and neuroinflammatory processes may coexist.

## Materials and methods

### Study sample

Data was obtained from 964 subjects from the subset of 1162 adults (initial sample) participating in the Barcelona Brain Health Initiative (BBHI: https://bbhi.cat/en/), a longitudinal cohort study investigating the determinants of brain and mental health in middle-aged and older individuals in which whom periodic cognitive, medical, brain imaging and biological assessments were performed.^26,54^ The main inclusion criteria in the BBHI were the absence of psychiatric or neurological medical diagnoses and being aged 40 or older. The specific inclusion criteria for the present study were (i) available brain MRI that met the quality check inspection requirements; (ii) normative neuroradiological reports (e.g. no brain tumour suspicions, stroke or moderate to severe white matter damage); and (iii) Mini-Mental State Examination (MMSE) equal or above 27 and a neuropsychological assessment without scoring below normative data on the administered tests given the age and educational status of the participants, as recommended elsewhere.^55,56^ A subset of participants underwent blood sampling for biomarker characterization (pTau181, NfL and hsCRP) and *APOΕ4* genotyping, as described below. The study was approved by the *Unió Catalana d’Hospitals* ethics committee (approval references: CEIC 17/06 and CEI 18/07) and the *Comissió de Bioètica de la Universitat de Barcelona*. Written informed consent was obtained from all participants in accordance with the Code of Ethics of the World Medical Association (Declaration of Helsinki).

### Neuropsychological assessment

A comprehensive battery of neuropsychological tests was administered (as described in Cattaneo *et al*.^26,56^) by expert neuropsychologists. Age- and education-adjusted Spanish norms were applied for all tests.^56^ As a measure of cognitive function, we computed the PACC, a composite including episodic memory, executive function and global cognition scores sensitive to early cognitive decline related to early Alzheimer’s disease pathology.^57,58^ We calculated *Z*-scores from valid tests representative of each mentioned domain, specifically the delayed trial of the Rey Auditory Verbal Learning Test (RAVLT-delayed), the animal semantic fluency test, the Symbol Digit Modalities Test (SDMT) and the MMSE, and then averaged them to obtain the final composite. Due to unavailability of data for specific cognitive tests for some subjects, the sample size with available PACC scores was *N* = 947 (from the final sample of *N* = 964 with usable MRI data—see next section).

### MRI data acquisition and preprocessing

MRI data were acquired in a 3 T Siemens scanner (MAGNETOM Prisma) with a 32-channel head coil, at the *Unitat d’Imatge per Ressonància Magnètica IDIBAPS (Institut d’Investigacions Biomèdiques August Pi i Sunyer)* at the *Hospital Clínic de Barcelona*, Barcelona. For all participants, MRI session included a high-resolution T1-weighted (T1w) structural image, obtained with a magnetization prepared rapid acquisition gradient-echo (MPRAGE) three-dimensional protocol and a total of 208 contiguous axial slices obtained in ascending fashion [repetition time (TR) = 2400 ms, echo time (TE) = 2.22 ms, inversion time = 1000 ms, flip angle = 8°, slice thickness = 0.8 mm and field of view (FOV) = 256 mm]. Additionally, a high-resolution three-dimensional SPACE T2-weighted (T2w) acquisition was undertaken [TR = 3200 ms, TE = 563 ms, flip angle = 120°, 0.8 mm isotropic voxel, FOV = 256 mm (208 total slices])]. Diffusion-weighted imaging was performed using a 99-direction multi-shell protocol with b-values ∼5, 1500 and 3000 s/mm². Images were acquired with 1.5 × 1.5 × 1.5 mm³ isotropic resolution, TR = 3230 ms and TE = 89.2 ms, using a multi-band echo planar imaging sequence (acceleration factor 4), with fat suppression. Images were reconstructed with magnitude reconstruction. All the MRI images were examined by a senior neuroradiologist to detect any clinically significant pathology. From an initial sample of 1162 subjects, a total of 61 were discarded in this step due to the presence of tumours, cysts, relevant micro-bleedings, cerebral arteriovenous malformations or other brain lesions. All the acquisitions were visually inspected before analysis to ensure that they did not contain MRI artefacts or excessive motion. In this visual quality control step, 10 subjects were excluded due to low quality of the image.

The FMRIB Software Library (FSL, version 5.0.11; https://fsl.fmrib.ox.ac.uk/fsl/fslwiki/)^59,60^ and FreeSurfer (version 6.0; https://surfer.nmr.mgh.harvard.edu/)^61^ were used for preprocessing diffusion weighted imaging (DWI) and structural MRI data, respectively. CTh was obtained from the recon-all FreeSurfer command, run with default parameters and the T2 flag to improve pial surface reconstruction. Regarding diffusion processing, the ‘eddy’ and ‘DTIFIT’ tools from FMRIB’s Diffusion Toolbox (FDT; FSL software) were used. For the processing of cMD, a homemade surface-based approach was implemented (available at www.gitlab.com/vmontalb/diffusion-on-surface), which is based on FreeSurfer algorithms and uses the Koo *et al*.^62^ partial volume correction toolbox (crucial in cMD calculations^63^). It can be found explained in detail in Montal *et al*.^21^Other homemade scripts were used to obtain regional and hemispherical mean cMD values. Of note, seven subjects were not fully processed due to unavailability of DWI data and therefore were not included in the final sample. The individual results were inspected visually in all cases to ensure accuracy of registration, skull stripping, segmentation and cortical surface reconstruction. At this point, 120 subjects were excluded due to erroneous preprocessing or low quality of the resulting image, leading to a final sample of 964.

### Plasma biomarkers and *APOΕ4* genotyping

Plasma pTau181, NfL and hsCRP quantifications were available for a subset of subjects (*N* = 668 for pTau181; *N* = 697 for NfL; *N* = 345 for hsCRP; *N* = 750 in total). Notice that data acquisition was independent for each plasma biomarker, therefore concluding in partially overlapping subsamples (that is, some participants have data for several markers). In the two former, blood samples were collected using ethylenediaminetetraacetic acid tubes during the medical assessment, and plasma was aliquoted and stored in a refrigerator at −80°C in a biobank facility following standard procedures usually employed for clinical purposes. The measurements were performed at the Clinical Neurochemistry Laboratory at the University of Gothenburg, Mölndal, Sweden, by board-certified laboratory technicians who were blinded to clinical data. pTau181 was measured using an in-house single molecule array (Simoa) method on an HD-X instrument (Quanterix, Billerica, MA, USA). Participants were labelled as ‘high’ when having pTau181 levels equal or above the 75th percentile (5.06 ng/mL), as has been done previously.^64^

Plasma NfL concentration was measured using the Simoa NF-light Advantage Kit on an HD-X instrument as described by the kit manufacturer (Quanterix, Billerica, MA). The study participants were determined to have high or low NfL levels according to the reference values,^65^ which consider different cut-offs at three age ranges: 10 pg/mL for ages < 50 years, 15 pg/mL for ages 50–60 years and 20 pg/mL for ages > 60 years.

For hsCRP quantification, plasma levels were measured by the AM-438 Quantification of CRP in Dried Blood Spots (DBS) following standard procedures, and analyses were performed on a MESO® QuickPlex SQ 120 Multiplex Imager using the V-PLEX Human CRP kit (K151STD-2; MSD, Rockville, Maryland, USA) as described in the manual. For hsCRP groups, the cut-off for high/low levels was set at 3 mg/L for all age ranges, as described in Bassuk *et al*.^36^

Regarding *APOΕ4*, data was available for *N* = 424 subjects, totalling to *N* = 853 subjects with biomarker data available (either pTau181, NfL, hsCRP or *APOE*). Buccal swabs were collected and genotyped using the Global Screening Array (GSA; Illumina, Inc. San Diego, CA, USA) with shared custom content, as performed previously and detailed elsewhere.^66,67^

### Statistical analyses

To characterize how cMD and CTh vary across the adult lifespan, vertex-wise analyses using a two-class general linear model (GLM) implemented in Freesurfer (‘mri_glmfit’ command) were performed. To better elucidate the relationship between variables and age, the sample was split into three age groups (AGs): AG1 (40–50 years; *N* = 300), AG2 (50–65 years; *N* = 484) and AG3 (>65 years; *N* = 180). GLM analyses were performed on each subsample for each cortical measure, and all analyses were adjusted by sex. Mean values for cMD and CTh obtained from homemade scripts were extracted to plot the results. When both cortical metrics were displayed together, dual *y*-axis plots with *Z*-scores were used to ensure comparability and scalability of the data. Some of these plots (Supplementary material) were restricted to regions of interest (ROIs) derived from subsequent analyses (see below).

To assess the relationship between cMD and CTh and other independent variables (i.e. PACC scores, pTau181, NfL and hsCRP concentrations and *APOE* ɛ4 status), a vertex-wise two-class GLM implemented in Freesurfer (‘mri_glmfit’) was used. All analyses were adjusted by age and sex, and years of education was introduced as another nuisance variable for PACC analyses to account for the potential confounding effect of educational level on the association between cognitive performance and other variables. Given that fluid biomarkers often exhibit non-linear relationships with brain characteristics, the sample was further split into high and low pTau181, NfL and hsCRP (see the cut-offs in the previous section), similarly as the *APOE* genotyping into ɛ4 carriers and non-carriers, to assess the differential effect of associations on risk profiles (Supplementary material). Additionally, analyses were also performed on a subsample with all biomarker data available (*N* = 242; Supplementary Table 1 and Fig. 2). Within-group differences were evaluated using the previously described GLM method for neuroimaging analyses. For non-neuroimaging group comparison analyses, mostly ordinary least squares linear regression was applied to account for the effect of sex and age, and additionally years of education for cognition measures, except in the following cases: (i) Student’s *t*-test was used in two-level group comparisons (risk profiles) for continuous uncorrected variables (age); (ii) a chi-square test in two- and three-level group comparisons (risk profiles and age groups) for categorical uncorrected variables (sex); (iii) an ANOVA in three-level group comparisons (age groups) for continuous uncorrected variables (age); and (iv) a logistic regression for two- and three-level group comparisons (risk profiles and age groups) for corrected categorical variables (*APOE*). Omnibus effects for linear and logistic regression models were evaluated using Type III sums of squares. Of note, age was not corrected for in age group comparison. Partial correlation coefficients were computed between the independent variables, adjusting all analyses by age (except when age was a predictor) and sex, and additionally years of education for PACC score regressions. Biomarker data were transformed depending on their distribution to meet model assumptions (normality) for the described analyses. For pTau181 a Box–Cox transformation was applied,^68,69^ while a log transformation was used for NfL and hsCRP.^70^

To investigate how cMD is associated with CTh, a correlation analysis between the two cortical integrity metrics was performed (i.e. cMD against CTh) using the ‘–pvr’ flag, which provides a custom regressor for each vertex across all vertices. The main analysis was uncorrected to observe the basal association between metrics and was repeated within age group subsamples in complementary analyses. To assess whether the relationship between the two integrity metrics is influenced by the levels of pathology-related or inflammation biomarkers, subsequent analyses were adjusted by age, sex and plasma biomarker levels. Additionally, ROIs derived from the results of these analyses were selected for dual *y*-axis plots correlating cMD and CTh with age for each ROI, as mentioned above.

For all neuroimaging analyses, the obtained maps were corrected for family-wise error (FWE) using a Monte Carlo Null-Z simulation, with 10 000 repetitions to avoid false positives, and only regions that survived those comparisons were considered. For visualization, scaled gamma values were represented onto a standard template semi-inflated surface, adjusting scaling and colour thresholds for each metric and contrast. For group statistics, maps were smoothed using a 2D Gaussian kernel of 15 mm full width at half maximum (FWHM). The resulting vertex-wise statistical maps were considered significant at *P* < 0.05 level. All non-neuroimaging statistical analyses were run in RStudio (version 4.4.1; 2024, The R Foundation for Statistical Computing).^71^

## Results

### Sample characterization

The final sample was composed of 964 participants, with an age range from 40 to 82 years and a balanced sex proportion (52% women). The sample’s demographic and biomarker characteristics are described in Table 1. Associations between the studied variables can be found in the Supplementary material (Supplementary Table 1), together with demographics by age group (Supplementary Table 3) and more details on biomarker subsamples (Supplementary Fig. 1).

**Table 1:** Study sample

		Whole sample *N* = 964	pTau181 subsample *N* = 646	NfL subsample *N* = 697	hsCRP subsample *N* = 344
Age [mean (SD)] *years*		56 (9.56)	54.29 (8.08)	53.4 (7.12)	53.27 (7.23)
Sex [*N* (%)]	Male	461 (47.8)	334 (50)	348 (50)	176 (51)
	Female	503 (52.2)	334 (50)	349 (50)	169 (49)
Education [mean (SD)] *years*		16.64 (3.79)	16.95 (3.7)	17.02 (3.61)	17 (3.64)
MMSE [mean (SD)]		29.68 (0.64)	29.76 (0.56)	29.8 (0.48)	29.82 (0.45)
PACC [mean (SD)]		0.001 (0.64)	0.025 (0.62)	0.047 (0.6)	0.05 (0.59)
pTau181	*N* (%)	668 (69)	668 (100)	646 (93)	275 (80)
	mean (SD) *pg/mL*	5.16 (7.81)	5.16 (7.81)	5.26 (7.93)	5.61 (8.36)
NfL	*N* (%)	697 (72)	646 (97)	697 (100)	291 (84.3)
	mean (SD) *pg/mL*	12.05 (4.65)	12.22 (4.69)	12.05 (4.65)	12.28 (4.66)
hsCRP	*N* (%)	345 (36)	275 (41)	291 (42)	345 (100)
	mean (SD) *mg/L*	1.21 (2.19)	1.18 (2.16)	1.23 (2.19)	1.21 (2.19)
*APOΕ ɛ4* status [*N* (%)]	Carriers	86 (20.3)	56 (20.1)	54 (19.8)	64 (21)
	Non-carriers	338 (79.7)	222 (79.9)	219 (80.2)	240 (79)

Continuous variables are displayed as mean (SD), while categorical variables are described as absolute numbers (frequency in %) for the whole sample and for each subsample of available plasma biomarker data. Given that each biomarker was acquired independently, as described in the Materials and Methods section, the availability of their data does not match for each participant, and samples are therefore partially overlapping.

SD, standard deviation; pTau181, phosphorylated tau 181; NfL, neurofilament light; APOΕ4, apolipoprotein ɛ4; hsCRP, high-sensitivity C-reactive protein.

### Associations between cortical integrity metrics and age

**Figure 1:**
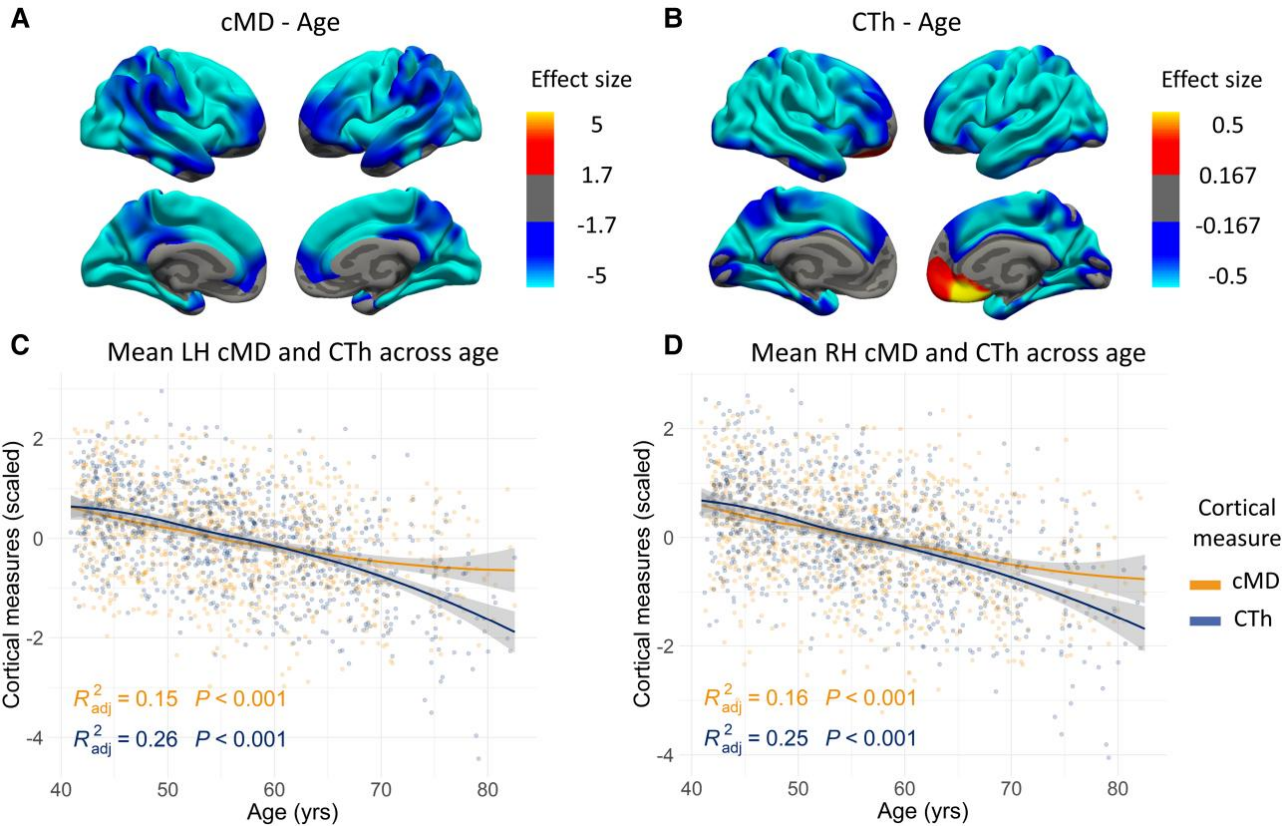
**Associations between cMD or CTh and age.** (**A**) Association between cMD and age. (**B**) Association between CTh and age. (**C**) Scatter plot of the association between mean cMD and CTh with age for the LH. (**D**) Scatter plot of the association between mean cMD and CTh with age for the RH. Sample size was *N* = 964 in all cases. A whole brain vertex-wise GLM was performed with brain measures (cMD or CTh) as the outcome variable and age as the predictor for **A** and **B**. Only clusters that maintained a *P*-value of <0.05 after FWE multiple comparison correction are shown. Thresholds were adjusted regarding the effect sizes (gamma values are scaled × 10^−7^ for cMD and × 10^−2^ for CTh) of the clusters for correct visualization. Scatter plots in **C** and **D** show the association of each metric (cMD in orange; CTh in blue) with age for the left and right hemispheres, respectively. For each cortical measure, the R-squared and *P*-value of the linear model covarying for sex are displayed, while the global trend is represented visually by LOESS method. In all cases, analyses are corrected for sex. cMD, cortical mean diffusivity; CTh, cortical thickness; LH, left hemisphere; LOESS, locally estimated scatterplot smoothing; FWE, family-wise error; *P*, *P*-value; R^2^adj, R^2^-adjusted; RH, right hemisphere; yrs, years.

**Figure 2:**
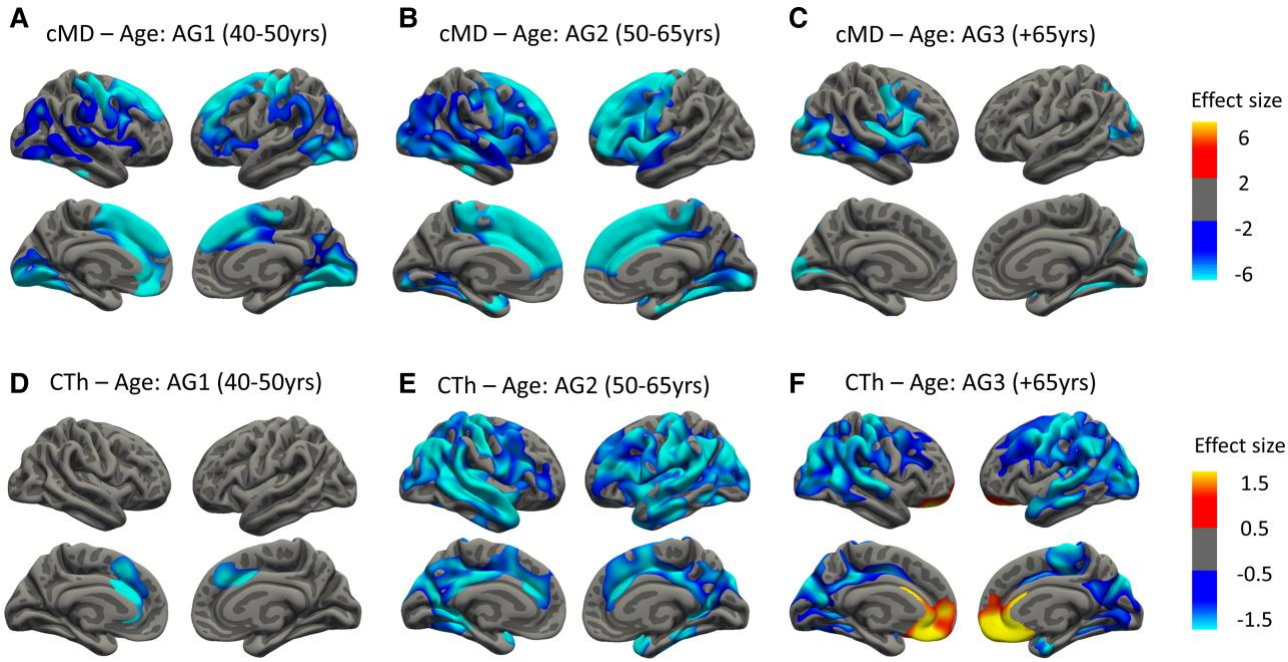
**Associations between cMD or CTh and age by age groups.** (**A–C**) Vertex-wise associations between cMD and age for AG1, 2 and 3, respectively. (**D–F**) Vertex-wise associations between CTh and age for AG1, 2 and 3, respectively. A whole brain vertex-wise GLM was performed with brain measures (cMD or CTh) as the outcome variable and age as the predictor, for each age group. All analyses are corrected for sex. Only clusters that maintained a *P*-value of <0.05 after FWE multiple comparison correction are shown. Thresholds were adjusted regarding the effect sizes (gamma values are scaled × 10^−7^ for cMD and × 10^−2^ for CTh) of the clusters for correct visualization. Age group subsamples were defined as AG1 including volunteers aged 40–50 (*N* = 300), AG2 including volunteers from 50 to 65 years old (*N* = 484) and AG3 including volunteers older than 65 years (*N* = 180). AG1, Age Group 1; AG2, Age Group 2; AG3, Age Group 3; cMD, cortical mean diffusivity; CTh, cortical thickness; FWE, family-wise error; yrs, years.

Vertex-wise GLM analyses showed a negative correlation with age for both cortical integrity metrics (i.e. cMD and CTh) (see Fig. 1; *N* = 964)), displaying a large cluster on both hemispheres mainly covering the whole cortex (Fig. 1A and B), except for a positive cluster in the right orbitofrontal region for CTh (Fig. 1B). Similar results were found in analyses on a subsample with all available biomarkers (*N* = 242; Supplementary Fig. 2). When repeating the same analysis by age groups, we identified that cMD clusters were more extensive at early ages (i.e. AG1 and AG2), while CTh clusters were larger in older groups (i.e. AG2 and AG3; see Fig. 2A–F). Age * group interactions showed that AG2 and AG3 differed in their associations between cMD and age in frontal and occipital regions (see Supplementary Fig. 3A). Linear models showed lower significance in the age correlation with cMD within AG3, while for CTh, the association was significant only in AG2 and AG3 (Supplementary Fig. 3B–G).

### Associations between cortical integrity metrics, cognition and biomarkers

**Figure 3:**
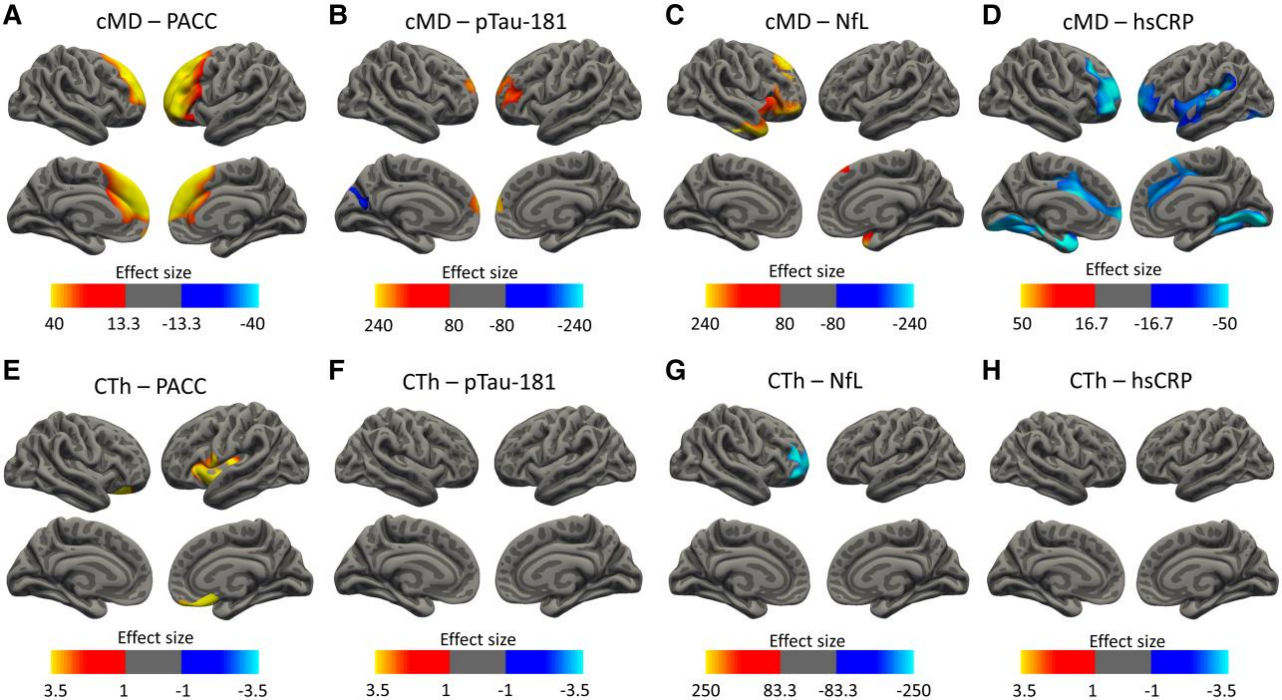
**Vertex-wise associations between cortical metrics and PACC scores, pTau181, NfL and hsCRP plasma concentrations.** (**A and E**) Associations between cMD and CTh with PACC, respectively (*N* = 947). (**B and F**) Associations between cMD and CTh with pTau-181, respectively (*N* = 646). (**C and G**) Associations between cMD and CTh with NfL, respectively (*N* = 697). (**D and H**) Associations between cMD and CTh with hsCRP, respectively (*N* = 344). A whole brain vertex-wise GLM was performed with brain measures (cMD or CTh) as the outcome variable and PACC scores, pTau-181, NfL or hsCRP plasma levels as predictors. All analyses are corrected for sex and age. Additionally, PACC analyses are corrected for years of education. Only clusters that maintained a *P*-value of <0.05 after FWE multiple comparison correction are shown. Thresholds were adjusted regarding the effect sizes (gamma values are scaled × 10^−7^ for cMD and × 10^−2^ for CTh) of the clusters for correct visualization. cMD, cortical mean diffusivity; CTh, cortical thickness; FWE, family-wise error; hsCRP; high-sensitivity C-reactive protein; NfL, neurofilament light; PACC, preclinical Alzheimer’s cognitive composite; pTau181, phosphorylated tau 181.

For cMD, a positive bilateral association with PACC scores was identified in a cluster comprising the frontal eye field, the dorsal anterior cingulate and anterior and dorsolateral prefrontal cortices (Fig. 3A; *N* = 947), while the association between CTh and PACC showed two smaller positive clusters in the pars opercularis, the pars triangularis and the anterior cingulate, insular and orbitofrontal cortices (Fig. 3E; *N* = 947). As in the previous section, we split the sample into age groups, and the results show positive cMD associations in larger areas at earlier ages (frontal clusters in AG1 and AG2) than CTh (temporal and parietal clusters in AG2 and AG3) (Supplementary Fig. 4).

Then, we examined the relationship between cortical integrity metrics and plasma biomarkers. For pTau181 (*N* = 668), a positive association with cMD was identified comprising a small frontopolar cluster and a small negative cluster in the visual cortices (Fig. 3B), while there were no significant results for CTh (Fig. 3F). Analyses on NfL (*N* = 697) showed positive right orbitofrontal and temporopolar clusters for cMD (Fig. 3C), and a right negative frontopolar cluster for CTh (Fig. 3G). Regarding hsCRP (*N* = 345), vertex-wise analyses showed negative clusters for cMD in the right dorsolateral prefrontal cortex, the left pars opercularis, the temporal pole, supramarginal and superior temporal gyri and auditory and gustatory cortices and bilateral frontopolar, anterior cingulate and visual cortices (Fig. 3D), while no significant results were drawn from the CTh analyses (Fig. 3H). Regarding *APOΕ4* analyses, we found lower cMD over a left temporal cluster in *APOΕ ɛ4* carriers compared to non-carriers (Supplementary Fig. 5D) and a negative cluster along the medial temporal lobe for the association between cMD and hsCRP levels only in non-carrier individuals (Supplementary Fig. 6).

As a complementary analysis, we tested whether individuals with worse biomarker characteristics (i.e. high plasma pTau181, NfL or hsCRP concentrations) or a genetic risk profile (i.e. *APOΕ ɛ4* carriers) present poorer cortical integrity values. In these analyses, we found higher cMD in frontal regions for those individuals with higher NfL levels compared to lower NfL levels (Supplementary Fig. 5B). For further details on risk profile comparison, see the Supplementary material (Supplementary Table 2 and Figs 5 and 7–10).

### Cortical integrity characterization: relationship between cMD and CTh

**Figure 4:**
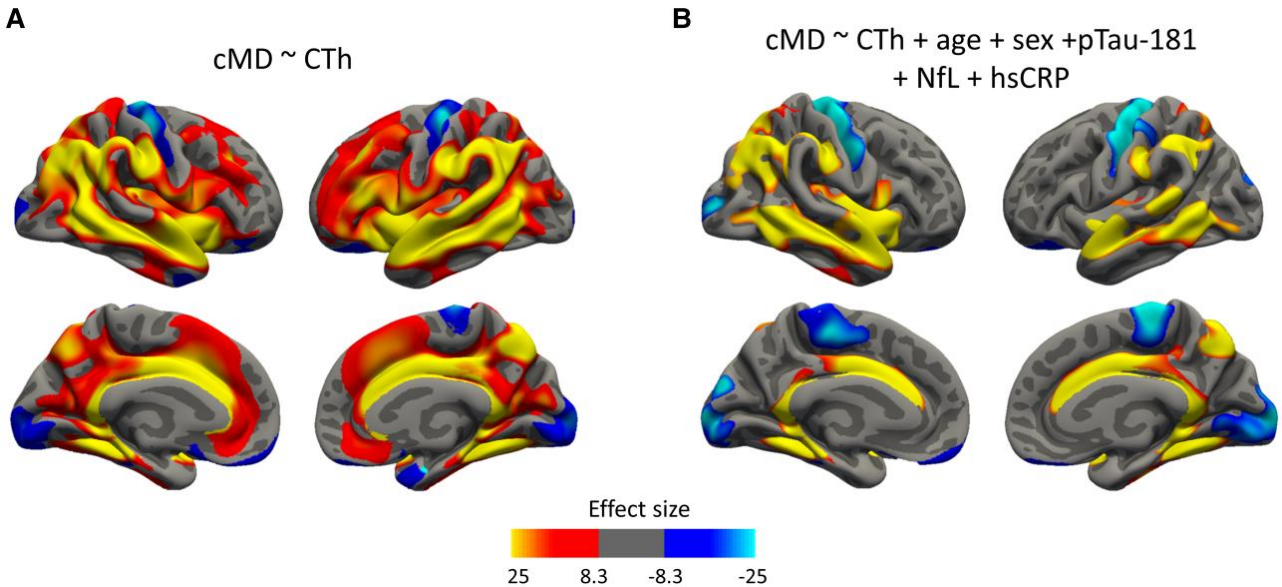
**Association between cMD and CTh.** (**A**) Uncorrected analysis only including cMD and CTh (*N* = 964). (**B**) Analyses correcting for age, sex and plasma biomarker levels (pTau-181, NfL and hsCRP) (*N* = 275). A whole brain vertex-wise GLM was performed with cMD as the outcome variable and CTh as the predictor in **A** and additionally covarying for age, sex, pTau-181, NfL and hsCRP levels in **B**. Only clusters that maintained a *P*-value of <0.05 after FWE multiple comparison correction are shown. Thresholds were adjusted regarding the effect sizes (gamma values are scaled × 10^2^) of the clusters for correct visualization. cMD, cortical mean diffusivity; CTh, cortical thickness; FWE, family-wise error; hsCRP; high-sensitivity C-reactive protein; NfL, neurofilament light; pTau181, phosphorylated tau 181.

Finally, we conducted vertex-wise analyses correlating cMD with CTh, and we obtained a significant positive cluster comprehending most of the cortex and some negative clusters in precentral, occipital, temporopolar and orbitofrontal areas (Fig. 4A; *N* = 964). This ‘pattern’ was maintained with minor changes and smaller clusters in subsequent analyses adjusting by age, sex and plasma biomarker levels (Fig. 4B; Supplementary Fig. 11), and similar results were also observed across age subsamples (Supplementary Fig. 12), with more restricted clusters.

## Discussion

Our results showed a widespread negative association between CTh and age, and the non-linearity and regionality of these changes, as has been extensively reported.^4,72-74^ The positive CTh–age association comprising the right orbital medial cortex has been reported previously,^75-77^ with diverse explanations linking it to healthy ageing,^77-79^ inflammation^76^ or cross-sectional artefacts.^77^ On the other hand, we observed a robust negative correlation between cMD and age across subsamples and analyses, suggesting it decreases along the lifespan. Importantly, splitting the analyses into age groups showed cMD associations in more extensive areas at ages 40–65 relative to older subjects, while the opposite was found for CTh, suggesting microstructural characteristics occur earlier than macrostructural changes. This has also been shown longitudinally in the Alzheimer’s disease continuum^80^ and in other neurological diseases such as essential tremor^81^ or aphasia.^82^

However, the available literature shows mostly increases in cortical diffusion metrics across the healthy adult lifespan in different regions, such as the hippocampus,^75,83^ prefrontal regions^84^ or the thalamus,^85^ and also in analyses comprehending broader areas.^86-91^ Furthermore, cMD increases have also been reported along the Alzheimer’s disease continuum both cross-sectionally^92-95^ and longitudinally.^80,96^ This is consistent with the general notion of tissue density reduction by means of extracellular space expansion and membrane reduction as a result from cellular component loss (dendrites, synapses, myelinated axons, glial cells, blood vessels, etc.),^88,97,98^ which has been verified to correspond to increased cMD in animal models^99^ and would ultimately lead to the cortical thinning observed in ageing.^84,90^ Our results do not seem to align with these views, but it is worth noting that not all previous studies may be directly comparable with ours, as many differ in sample size and characteristics, design and specific methods, and pathological progression is often considered rather than age. We speculate our observations might be supported by more membranous cellular structures relative to free water spaces, or due to a shift in the concentration of extracellular and intracellular water resulting in a denser organization. Indeed, a recent study using soma and neurite density imaging (SANDI), a multi-compartmental diffusion MRI model that provides soma and neurite signal fractions,^100^ observed a similar unexpected result, with the intra-neurite diffusivity fraction increasing along the lifespan, perhaps indicating neurite’s orientation and packing changes leading to denser structures and increased anisotropic diffusion patterns (or resulting from technical biases related to SANDI).^14^ Another possible concomitant cellular phenomenon affecting our result is that of increased immunological activity, as discussed below. Other histopathological studies indicate how subtle the changes detected by MD in GM are, corresponding to axon directionality alterations in specific cortical layers, rather than dendritic arborization or synaptic changes.^101^ Additionally, we cannot discard cell replacement related to tissue repair and astrogliosis following neuronal loss, as known to occur in ageing and neurodegenerative diseases^102,103^ and influence microstructure and diffusion MRI signal.^104^ Overall, several biological processes might be contributing to the observed results, potentially showing a discordant cMD effect characteristic of ageing, and perhaps discernible with other diffusion MRI models; as pointed elsewhere, we cannot determine a single underlying cause of microstructural change.^16,96^ In this regard, and considering the novelty of the metric used, these results would strongly benefit from replication in an independent sample in future work.

We saw a negative correlation between cognition (i.e. PACC scores) and age, as expected and previously reported,^80,105^ although other studies find stability in this composite.^106-108^ We also observed lower CTh was associated with lower cognition, as has been previously reported,^109,110^ and for specific cognitive domains such as executive function,^111^ associative recognition^112,113^ and general cognition^113,114^ and using longitudinal approaches.^113^ On the other hand, we saw a positive correlation between cMD and PACC scores in extensive prefrontal regions. Furthermore, when splitting the sample into age groups, the results suggest cMD can detect changes relevant to cognitive performance earlier than CTh. Indeed, this metric has been previously proposed as highly specific to cognitive change.^96,115,116^

Regarding the direction of the association, mostly the reported results on ageing are negative associations, found in several areas such as the medial temporal lobe and the hippocampus,^23,84,117-119^ the precuneus,^87^ the prefrontal cortex (PFC)^89,115^ or other areas within the default mode network,^116^ and are supported by longitudinal approaches^24,96^ and mediation analyses,^80^ generally concluding reduced microstructural integrity impairs cognition. However, some studies do find a positive correlation between cMD and empathizing,^120^ emotional intelligence^121^ and cognitive performance.^122^ We hypothesize this could be reflective of inflammation and swelling reduction or glial-related synaptic pruning processes, as has been argued before,^122^ although in a particular context (physical exercise intervention on mild cognitive impairment patients). The two former cited studies report results in diverse structures including the PFC, a region subject to many structural changes in ageing,^123^ perhaps some contributing in generating a denser sub-optimal environment for signal processing,^124^ and others related to plasticity mechanisms^125^ leading to frontal circuitry optimization, in line with the hypothesis of posterior-to-anterior shift in functional compensation.^78,126^ Additionally, the relationship between cognition and cMD is probably different across cognitive domains and influenced by external factors.^116^

We found positive correlations between pathology-related plasma biomarkers (pTau181 and NfL) and age, both of which have been reported in the literature,^65,127,128^ but failed to replicate it for hsCRP, perhaps due to the generally low hsCRP levels in our healthy population, although other studies have seen such correlations in similar samples.^129^ cMD presented a clear positive association with pTau181 and NfL in frontal regions and negative associations on more widespread areas for hsCRP, possibly reflecting neurodegenerative and inflammation-related processes, respectively. In the literature, this has only been clearly reported for NfL in the Alzheimer’s disease continuum,^96,130^ while pTau181^131^ and hsCRP^44^ have only been related to MD in WM. However, higher peripheral CRP has been associated with decreased cMD in animal studies^132^ and with diminished intracortical WM in humans, a tissue that may affect cMD,^44^ and has been argued as possibly altering fundamental biological processes underlying water diffusivity.^133^ Crucially, CTh presented a negative association with NfL, as previously reported,^134,135^ but no results were found for the other biomarkers. However, in the literature, results are mixed for pTau181^64,134,135^ but in general negative associations are reported between hsCRP and CTh.^129,136,137^

These results present cMD as a more sensitive measure than CTh, and they highlight microstructural alterations as prior occurrences to macrostructural changes. Moreover, the diverging associations of both neuroimaging metrics for NfL, a marker for neuronal damage,^138,139^ could indicate a predominantly neurodegenerative environment, as postulated in the later phases of the biphasic model proposed by Montal *et al*.^21^ This has been seen along the Alzheimer’s disease continuum in recent studies,^96^ but perhaps is also characteristic of age-related processes, to which frontal regions are especially vulnerable.^53,140^ Similarly, pTau181 associations in prefrontal areas could be related to amyloid deposition,^95^ known to occur early in those regions,^141-143^ and seen related to higher diffusivity in GM,^144^ although this is under debate.^145^ However, in cognitively unimpaired adults, these observations might also be detecting frontal detrimental processes intrinsic to ageing but not necessarily causally related to pathology progression.^53,146^ Thus, the results underline the joint use of cMD together with more ‘traditional’ neuroimaging metrics (e.g. CTh) as capable to discern pathological trajectories and their underlying phenomena.

Importantly, some regions highlighted in hsCRP associations seem spared in pTau181 and NfL results (namely in the PFC, anterior cingulate, lingual and fusiform cortices and left insular and supramarginal cortices), and we hypothesize inflammatory-related phenomena might be occurring in those areas. In the literature, insular,^147^ superior temporal,^147^ supramarginal,^129,148^ anterior cingulate,^129^ parahippocampal and entorhinal^129^ cortices’ atrophy has been related to peripheral inflammation markers. The role of peripheral inflammation and its effect on brain structure has been increasingly acknowledged in ageing,^19,44,149,150^ neurodegenerative diseases^151-153^ and Alzheimer’s disease pathophysiology,^154,155^ even at low levels.^66^ The general notion is that peripheral inflammation may alter blood–brain barrier properties, facilitating immune-factor penetrance into the central nervous system and activating local glia and microglia.^44,45,149^ We consider this cascade could result from low-grade chronic alterations in the immune system and constitute a possible risk factor for Alzheimer’s disease,^156^ but being overall a mechanism intrinsic of ageing compatible with longevity,^19^ and perhaps independently affecting neurological function.^157,158^ This offers a complex picture in which effects could be generalized rather than effect specific regions, since vascular and genetic changes related to immune response are widespread.^159^ Interestingly, an animal study finds decreased MD in the hippocampus together with increased serum CRP levels after a whole-body radiation exposure.^132^ Furthermore, glial activation has been previously shown to alter diffusion properties of the cortical tissue,^160^ and decreased cMD has been linked to gliosis and microglial activation in mice models^161,162^ and to astrocytosis in humans,^163^ evidencing the suitability of this metric to detect central inflammatory processes. Therefore, our results reveal cMD as especially sensitive to cortical inflammatory processes and point to a relationship between peripheral and central inflammation.

Crucially, prefrontal regions seem affected in all plasma biomarker associations, which we interpret as indicating differential phenomena to which the PFC is regarded as especially vulnerable. Specifically, we might be observing possible early amyloid deposition or detrimental changes for pTau181, early neurodegeneration or plasticity-related changes for NfL, and a neuroinflammatory response for hsCRP.^45^ All these biological events might not be exclusive from one another (e.g. systemic inflammation has been related to structural degeneration^45^) and are probably occurring in a regionally distinct manner, with other factors such as lifestyle and genetic background likely influencing their variability and relative dominance in the cellular environment, which overall shows restricted water diffusivity across the lifespan. In summary, these plasma biomarker results could be pointing to incipient pathology-related but not deterministic mechanisms, perhaps concomitant in ageing at low levels, and presenting a risk factor when elevated.

Regarding *APOE*, some authors suggest a greater accumulation of low-level inflammatory stress throughout the lifespan in *APOΕ ɛ4* carriers as a mechanism for Alzheimer’s disease development.^75,164^ This hypothesis is supported by several mechanistic explanations involving inflammation modulation,^165-168^ lipid metabolism^169^ and blood–brain barrier integrity^170^ and may align with our results showing lower cMD in anterior lateral temporal regions in carriers compared to non-carriers. Furthermore, our findings on the significantly more negative relationship between cMD and hsCRP in temporal and frontal regions for non-carriers might indicate different responses to peripheral inflammation, but results should be interpreted with caution considering the lack of specificity of this biomarker.^155^ In line with this, other studies have seen microstructural alterations between carriers and non-carriers using other diffusion measures,^50-52^ although the specific implications remain unclear. Specifically, one study reports an interaction effect between *APOE* status and age, suggesting age-dependent changes are accelerated in carriers.^52^

Lastly, we correlated both cortical metrics between them (that is, cMD and CTh), identifying both negative and positive associations spanning across somatosensory and association areas, respectively. This could be reflective of the different architectonic and cellular properties of each region. However, studies on the Alzheimer’s disease continuum relating CTh and cMD found widespread negative correlations,^22,90^ linking thickness reduction and diffusion increases to the same integrity loss processes. Nonetheless, studies on ageing and preclinical subjects indicate the complementarity of both metrics when finding cMD associations with disease progression after correcting for CTh,^22,24^ or the independence of macrostructural and microstructural processes when observing cMD results remaining intact after correcting for CTh.^22,24,96^ Interestingly, one study finds such associations only in Aβ-negative individuals,^95^ possibly indicating different processes occurring in ageing and Alzheimer’s disease. However, our results were similar after correcting for biomarker levels and across age groups, perhaps because of the absence of pathology in our sample, and may be indicative of different susceptibilities of each region to damage. To make results more comparable and further clarify this point, further examination on risk profiles (e.g. *APOΕ4* status and high versus low biomarker levels) is recommended.

Regarding the limitations of the present study, we used a cross-sectional design, which can overestimate the age effect^73^ and be subject to cohort bias, which might contribute in the CTh increases seen in prefrontal regions. Longitudinal designs allow within-subject change verification, rather than only showing lifelong interindividual differences.^171-173^ However, we have a large sample size and a considerable age range, which adds statistical power and a bigger scope to detect age-related changes. Despite this, as mentioned above, our results would certainly benefit from independent-sample replication.

Also, it is worth keeping in mind that DTI-derived measures have limited biological specificity due to the assumptions of the model,^174^ especially when representing complex multicellular events,^175^ as multiple physiological^16^ and genetic factors^90,176^ influence their variation. Indeed, MD is sometimes regarded as having especially little reliability among diffusion measures,^177^ and therefore interpretations should be cautious, considering promising alternative diffusion-based models are on the rise.^100,178-182^ Nonetheless, we and others (i.e. Spotorno *et al*.^96^ and Sun *et al*.^80^) have shown its sensitivity to processes related to cognitive decline. Furthermore, the simplicity, applicability and comparability of DWI-derived metrics make them promising tools for the non-invasive study of microstructural changes associated with pathology in clinical practice.^17,183-187^ Nonetheless, open-access and standardized pipelines for cMD calculation are lacking, hindering its direct comparability among studies.

It is also relevant to mention the global nature of plasma biomarkers, as opposed to spatial MRI results. However, spatially accurate biomarker data (i.e. PET) would convey different information, and our multimodal approach also allows interpretation of possible biological substrates of microstructure alterations.^16^ Another related limitation is the lack of plasma samples for all participants; despite this, blood sample availability was considerable (*N* = 853). On the other hand, although plasma pTau217 has proved to be more sensitive to preclinical stages than other plasma tau variants,^188-190^ pTau181 is nonetheless sensitive to clinical progression across populations^191^ and amyloid deposition over time,^192,193^ and still regarded as a relevant marker.^139^ Lastly, it should be noted that NfL and hsCRP are biomarkers reflective of complex and central processes common in many neurological diseases;^152,194^ however, they show great versatility and clinical potential, specially NfL.^138^

## Conclusions

We showed that cMD is sensitive to microstructural changes related to neurodegeneration and neuroinflammatory phenomena occurring prior, complementarily and possibly (up to some degree and in a region-dependent manner) independently to macrostructural changes and pathology-related processes. We report this metric decreasing with age in our sample, suggesting cortical density increases in the adult lifespan, possibly resulting from multiple events related to cortical organization changes (i.e. tissue replacement, detritus accumulation, plasticity changes, inflammation and neurodegeneration) reflecting an effect on cortical diffusivity characteristic of ageing, which further longitudinal studies should aid to clarify. Furthermore, cMD seems to be especially more sensible than CTh to cognitive performance and neuroinflammation, which highlights its potential role in transversal clinical settings.

## Supplementary Material

fcaf511_Supplementary_Data

## Data Availability

The data that support the findings of this study are not publicly available due to participant privacy and ethical restrictions, but anonymized data may be shared by the corresponding author upon reasonable request and with appropriate institutional approvals. All neuroimaging preprocessing and analysis were performed using FreeSurfer (https://surfer.nmr.mgh.harvard.edu/) and FSL (https://fsl.fmrib.ox.ac.uk/), which are freely available for academic use. In-house analysis scripts and custom code used for data processing are available at the following repositories: www.gitlab.com/vmontalb/diffusion-on-surface and https://github.com/operera-cruz/cMD-CTh.
