# Supplementary material for: Cortical mean diffusivity detects early age-related changes and associates with cognition and plasma biomarkers
Source: Brain Commun. 2026 Jan 10;8(1):fcaf511. doi: 10.1093/braincomms/fcaf511 (PMC12933213; doi:10.1093/braincomms/fcaf511)
Supplement: fcaf511_Supplementary_Data [file fcaf511_supplementary_data.docx]

**Supplementary Material for:**

Cortical mean diffusivity detects early age-related changes and associates with cognition and plasma biomarkers

Short title: Cortical diffusivity detects age changes

Oriol Perera-Cruz^1,2^, Cristina Solé-Padullés^1,2^, Lídia Mulet-Pons^1,2^, María Cabello-Toscano^1,2^, Rachel M. Morse^1,2^, Kilian Abellaneda-Pérez^3,4,5^, Rubén Perellón-Alfonso^2,3,5,6,7^, Gabriele Cattaneo^3,5^, Javier Solana Sánchez^3,5^, Vanessa Alviarez-Schulze^3,5^, Nuria Bargalló^1,2,7,8^, Juan Fortea^9,10^, Jose M Tormos^3,11^, Alvaro Pascual-Leone^12,13^, Henrik Zetterberg^14,15,16,17,18,19,20,21^, Lídia Vaqué-Alcázar^1,2,3^*, David Bartrés-Faz^1,2,3^*

*^1^ Department of Medicine, Faculty of Medicine and Health Sciences and Institute of Neurosciences, University of Barcelona, Barcelona, Spain*

*^2^ August Pi I Sunyer Institute of Biomedical Research (IDIBAPS), Barcelona, Spain*

*^3^ Guttmann Institute, Institut Universitari de Neurorehabilitació adscrit a la Universitat Autònoma de Barcelona, Badalona, Spain*

*^4^ Universitat Autònoma de Barcelona, Bellaterra (Cerdanyola del Vallès), Spain*

*^5^ Fundació Institut d’Investigació en Ciències de la Salut Germans Trias i Pujol, Badalona, Spain*

*^6^ Unit for Cognitive Neuroscience, Institute of Neurosciences, University of Barcelona, Barcelona, Spain*

*^7^ Centre for Biomedical Research on Mental Health (CIBERSAM), Instituto de Salud Carlos III, Madrid, Spain*

*^8^ Neuroradiology Section, Radiology Department, Diagnostic Image Center, Hospital Clinic of Barcelona, University of Barcelona, Barcelona, Spain*

*^9^ Sant Pau Memory Unit, Department of Neurology, Institut d’Investigacions Biomèdiques Sant Pau- Hospital de Sant Pau, Barcelona, Spain*

*^10^ Barcelona Down Medical Center, Fundació Catalana de Síndrome de Down, Barcelona, Spain*

*^11^ Centro de Investigación Traslacional San Alberto Magno, Universidad Católica de Valencia San Vicente Mártir, València, Spain*

*^12^ Hinda and Arthur Marcus Institute for Aging Research and Deanna and Sidney WolkCenter for Memory Health, Hebrew SeniorLife, Harvard Medical School, Boston, MA, USA*

*^13^ Department of Neurology, Harvard Medical School, Boston, MA, USA*

*^14^ Department of Psychiatry and Neurochemistry, Sahlgrenska Academy, Institute of Neuroscience and Physiology, University of Gothenburg, Gothenburg, Sweden*

*^15^ Department of Neurodegenerative Disease, UCL Institute of Neurology, London, UK*

*^16^ Clinical Neurochemistry Laboratory, Sahlgrenska University Hospital, Mölndal, Sweden*

*^17^ Department of Pathology and Laboratory Medicine, University of Wisconsin School of Medicine and Public Health, Madison, WI, USA*

*^18^ Wisconsin Alzheimer’s Disease Research Center, University of Wisconsin School of Medicine and Public Health, University of Wisconsin-Madison, Madison, WI, USA*

*^19^ UK Dementia Research Institute at UCL, London, UK*

*^20^ Hong Kong Center for Neurodegenerative Diseases, InnoHK, Hong Kong, China*

*^21^ Centre for Brain Research, Indian Institute of Science, Bangalore, India*

*Corresponding authors:

Lídia Vaqué-Alcázar, email address: [lidiavaque@ub.edu](mailto:lidiavaque@ub.edu)

David Bartrés-Faz, email address: [dbartres@ub.edu](mailto:dbartres@ub.edu)

Postal address: Universitat de Barcelona, Facultat de Medicina i Ciències de la Salut, Carrer Casanova, 143, 08036 Barcelona, Spain; Telephone: +34 934 039 295

**Associations between the studied variables**

When examining the associations between the studied variables, partial correlations revealed that PACC significantly decreased with age (r=-0.385, p<0.001), while pTau181 and NfL were significantly associated with age (pTau181: r=0.147, p<0.001; NfL: r=0.492, p<0.001), and hsCRP only presented a trend towards significance (r=0.089, p=0.096). Also, both pathology-associated biomarkers were positively correlated between them (r=0.176, p<0.001), but only pTau181 presented a positive correlation with hsCRP (r=0.153, p=0.011). The results were similar when correlations were applied in the subsample with all plasma biomarker data available (Supplementary Table 2).

| Full sample (N=964) | | | | | | | | |
| --- | --- | --- | --- | --- | --- | --- | --- | --- |
|  | PACC | | pTau-181 | | NFL | | hsCRP | |
|  | r | p | r | p | r | p | r | p |
| Age | -0.385* | <0.001 | 0.147* | <0.001 | 0.492* | <0.001 | 0.089 | 0.096 |
| PACC | 1 | - | 0.006 | 0.877 | 0.001 | 0.970 | -0.053 | 0.327 |
| pTau181 |  |  | 1 | - | 0.176* | <0.001 | 0.153* | 0.011 |
| NFL |  |  |  |  | 1 | - | -0.007 | 0.906 |
| hsCRP |  |  |  |  |  |  | 1 | - |
| All plasma biomarkers subsample (N=242) | | | | | | | | |
|  | PACC ​ | | ​ pTau-181 ​ | | NfL | | hsCRP ​ | |
|  | r ​ | p ​ | r ​ | p ​ | r ​ | p ​ | r ​ | p ​ |
| Age ​ | -0.413* ​ | <0.001 ​ | 0.207* ​ | <0.001 ​ | 0.538* ​ | <0.001 ​ | 0.112 ​ | 0.082 ​ |
| PACC ​ | 1 ​ | - ​ | 0.057 ​ | 0.377 ​ | 0.007 ​ | 0.912 ​ | <-0.001 ​ | 0.996 ​ |
| pTau181 ​ | ​ | ​ | 1 ​ | - ​ | 0.195* ​ | 0.002 ​ | 0.189* ​ | 0.003 ​ |
| NFL ​ | ​ | ​ | ​ | ​ | 1 ​ | - ​ | -0.012 ​ | 0.855 ​ |
| hsCRP ​ | ​ | ​ | ​ | ​ | ​ | ​ | 1 ​ | - ​ |

**Supplementary Table 1.** **Correlations between independent variables for the whole sample *(above; N=964)* and the subsample with all plasma biomarkers available *(below, N=242)****. Pearson correlation coefficients are shown for the correlation between each independent variable. All partial correlations are adjusted for sex and age, except when age is the main variable. Additionally, PACC correlations are also adjusted by years of education. R coefficients corresponding to significant (<0.05) values are indicated with an asterisk (*). Abbreviations: hsCRP; high-sensitivity C-reactive protein; NfL, neurofilament light; PACC, Preclinical Alzheimer’s Cognitive Composite; pTau-181, phosphorylated tau 181.*

**Risk profile groups: pTau-181, NfL, hsCRP, and APOE4**

When comparing risk and non-risk profiles, we found that individuals classified within the high pTau-181 presented higher proportion of females and higher education years, and also significantly higher NfL levels (Supplementary Table 2). Individuals in the NfL group showed significantly higher PTau-181 levels, as well as individuals in the high hsCRP group (Supplementary Table 2). Regarding APOE, we found lower hsCRP levels among APOE4 carriers (t=-3.226, p=0.001) (Supplementary Table 2). Of note, none of the groups differed in age nor sex distribution except for NfL, in which individuals with higher levels were significantly older.

Regarding the vertex-wise analyses, none of the group’s comparisons yielded any differences regarding CTh. On the other hand, we identified no cMD differences in high pTau-181 individuals compared to those with lower levels (Supplementary Figure 3A) and likewise for hsCRP (Supplementary Figure 3C). However, we found higher cMD in frontal regions for those individuals with higher NfL levels (Supplementary Figure 3B), and APOE4 carrier individuals compared to non-carriers exhibited less cMD over a left temporal cluster (Supplementary Figure 3D).

For ApoE4, no differences were found between carriers and non-carriers regarding the association of each metric with hsCRP levels. However, when analyzing the subsamples, a negative cluster along the medial temporal lobe was found for the association between cMD and hsCRP levels only in non-carrier individuals (Supplementary Figure 4). No results were found for CTh associations (data not shown).

Additionally, trajectories for both cortical integrity metrics along the adult lifespan were reported for selected areas with the available sample split by: (i) pTau-181 groups (Supplementary Figure 5), (ii) NfL groups (Supplementary Figure 6), (iii) hsCRP groups (Supplementary Figure 7), and (iv) APOE status (Supplementary Figure 8). The chosen regions of interest were derived from the pattern obtained in the cMD-CTh analyses (medial orbitofrontal, middle temporal, posterior cingulate, rostral anterior cingulate, pericalcarine and precentral cortices; see Figure 4.

|  | pTau-181 | | | | NfL | | | |
| --- | --- | --- | --- | --- | --- | --- | --- | --- |
|  | High  N=166 | Low  N=480 | stat | p-value | High  N=177 | Low  N=520 | stat | p-value |
| Age  [mean (SD)] - years | 54.7 (7.25) | 53.2 (7.09)​ | -2.334 | 0.02* | 51.63 (7.11) | 53.99 (7.03) | 3.832 | <0.001* |
| Sex  [N (%)] - females | 72  (44.4) | 262 (52.3) | 3.234 | 0.07 | 86  (48.6) | 263 (50.6) | 0.137 | 0.711 |
| Education  [mean (SD)] - years | 17.5 (3.45) | 16.9 (3.69) | 4.643 | 0.032* | 16.89 (3.54) | 17.07 (3.64) | 0.729 | 0.394 |
| MMSE  [mean (SD)] | 29.81 (0.48) | 29.79 (0.49) | 1.121 | 0.862 | 29.82 (0.47) | 29.79 (0.49) | 0.163 | 0.687 |
| PACC  [mean (SD)] | -0.01 (0.59) | 0.06 (0.61) | 0.797 | 0.372 | 0.11​ | 0.03 | 0.108 | 0.742 |
| pTau181  [mean (SD)] - ng/mL | 10.9 (14.2) | 3.31 (0.736)​ | - | - | 5.406 (4.63) | 5.204 (8.79) | 7.904 | 0.005* |
| NFL  [mean (SD)] - ng/mL | 13.8 (5.46) | 11.7 (4.27)​ | 16.758 | 4.79e-5​* | 16.229 (5.57) | 10.628 (3.24) | - | - |
| hsCRP  [mean (SD)] - mg/L | 1.36 (1.92) | 1.11 (2.24) | 1.911 | 0.168 | 1.428 (2.31)​ | 1.156 (2.15) | 0.028 | 0.866 |
| APOE4  [N (%)] - carriers | 9 (13.9%) | 47 (22.1%)​ | 2.14 | 0.143 | 16 (22.2%) | 38 (18.9%) | 0.3 | 0.584 |

**Supplementary Table 2*.* Demographics by risk groups.**

|  | hsCRP | | | | APOE4 | | | |
| --- | --- | --- | --- | --- | --- | --- | --- | --- |
|  | High​  N=29 | Low  N=315​ | stat​ | p-value ​ | Carrier  N=86 | Non-carrier  N=338 | stat | p-value |
| Age  [mean (SD)] - years | 53.80 (7.41)​ | 53.22​ (7.22) | -0.408 | 0.686 | 55.99 (10.8) | 58.48 (10.6)​ | 1.907 | 0.059 |
| Sex  [N (%)] - females | 175  (51)​ | 169 (49)​ | 0​ | 1​ | 46  (53.5) | 180 (53.1) | <0.001 | 1 |
| Education  [mean (SD)] - years | 16.31​ (3.29) | 17.07​ (3.67)​ | 1.061 | 0.304 | 15.97 (3.91) | 16.39 (3.89) | 2.825 | 0.094 |
| MMSE  [mean (SD)] | 29.76 (0.51) | 29.82 (0.45) | 0.331 | 0.566 | 29.63 (0.64) | 29.66 (0.69) | 1.659 | 0.199 |
| PACC  [mean (SD)] | -0.04 (0.53) | 0.06 (0.6) | 0.375 | 0.541 | 0.02  (0.64) | -0.05 (0.67) | 0.136 | 0.712 |
| pTau181  [mean (SD)] - ng/mL | 8.38​ (7.51) | 5.34​ (8.40) | 5.814 | 0.0167* | 7.22  (17.9) | 5.65 (9.24)​ | 3.475 | 0.063 |
| NFL  [mean (SD)] - ng/mL | 13.83​ (6.93) | 38.03​ | 0.904 ​ | 0.343​ | 11.69 (4.09) | 12.31 (4.75)​ | 0.038 | 0.846 |
| hsCRP  [mean (SD)] - mg/L | 7.03​ (3.91) | 0.67 (0.65)​ | - | - | 1.12  (2.35) | 1.22 (2.00) | 10.733 | 0.001* |
| APOE4  [N (%)] - carriers | 64 (21%)​ | 240 (79%)​ | 0.523 | 0.469 | - | - | - | - |

**Supplementary Table 2. Demographics by risk groups.** *For each risk group subsample, demographics as in Table 1 are shown. Continuous variables are displayed as mean (SD), while categorical variables as absolute numbers (frequency in % within the sample). Statistical testing was performed as detailed in the Methods section, and the statistics and p-values for each test are displayed. Specifically, t for Student’s t-test, X^2^ for chi-squared and F for ANOVA are reported. Significant tests (p-value < 0.05) are indicated with an asterisk (*). Abbreviations: APOE4, apolipoprotein e4; hsCRP; high-sensitivity C-reactive protein; NfL, neurofilament light; MMSE, Mini-Mental State Examination; PACC, Preclinical Alzheimer’s Cognitive Composite; pTau-181, phosphorylated tau 181.*

|  | Age Groups | | |  |  |
| --- | --- | --- | --- | --- | --- |
|  | Age group 1  (40 – 50 yrs)  N=300 | Age group 2  (50 – 65 yrs)  N=484 | Age group 3  (>65 yrs)  N=180 | stat | p-value |
| Age [mean (SD)] - years | 45.34 (2.28) | 57.08 (4.19) | 70.86 (4.47) | 5158 | <0.001* |
| Sex [N (%)] - females | 144 (48) | 241 (49) | 118 (66) | 16.11 | <0.001* |
| Education [mean (SD)] - years | 17.32 (3.38) | 17.04 (3.89) | 14.42 (3.41) | 38.586 ​ | <0.001* |
| MMSE [mean (SD)] | 29.86 (0.41) | 29.74 (0.55) | 29.2 (0.93) | 52.55 | <0.001* |
| RAVLT [mean (SD)] | 54.84 (7.97) | 50.32 (8.46) | 50.84 (9.07) | 29.152 | <0.001* |
| Semantic fluency [mean (SD)] | 25.09 (4.65) | 23.67 (5.09) | 20.55 (4.52) | 36.751 | <0.001* |
| SDMT [mean (SD)] | 82.76 (12.73) | 75.56 (12.06) | 47.43 (13.96) | 404.751 | <0.001* |
| PACC [mean (SD)] | 0.31 (0.49) | -0.06 (0.60) | -0.38 (0.73) | 61.306 | <0.001* |
| pTau181 [mean (SD)] - ng/mL | 5.03 (8.39) | 5.36 (7.83) | 5.7 (5.39) | 8.972 | <0.001* |
| NfL [mean (SD)] - pg/mL | 9.73 (3.18) | 13.08 (4.71) | 16.94 (5.3) | 81.659 | <0.001* |
| hsCRP [mean (SD)] - mg/L | 1.15 (2.10) | 1.21 (2.19) | 1.65 (2.76) | 0.61 | 0.544 |
| APOE4 [N(%) – carriers] | 33 (29) | 32 (17) | 21 (18) | 6.459 | 0.039* |

**Supplementary Table 3. Demographics by age groups.** *For each age group subsample, the same demographics as in Table 1 are shown. Continuous variables are displayed as mean (SD), while categorical variables are described as absolute numbers (frequency in % within the sample). Statistical testing was performed as detailed in the Methods section, and the statistics and p-values for each test are displayed. Specifically, X^2^ for chi-squared and F for ANOVA are reported. Significant tests (p-value < 0.05) are indicated with an asterisk (*). Abbreviations: APOE4, apolipoprotein e4; hsCRP; high-sensitivity C-reactive protein; NfL, neurofilament light; MMSE, Mini-Mental State Examination; PACC, Preclinical Alzheimer’s Cognitive Composite; pTau-181, phosphorylated tau 181; RAVLT, Ray Auditory Verbal Learning Test; SDMT, Symbol-Digit Modalities Test.*

**Biomarker availability subsamples**


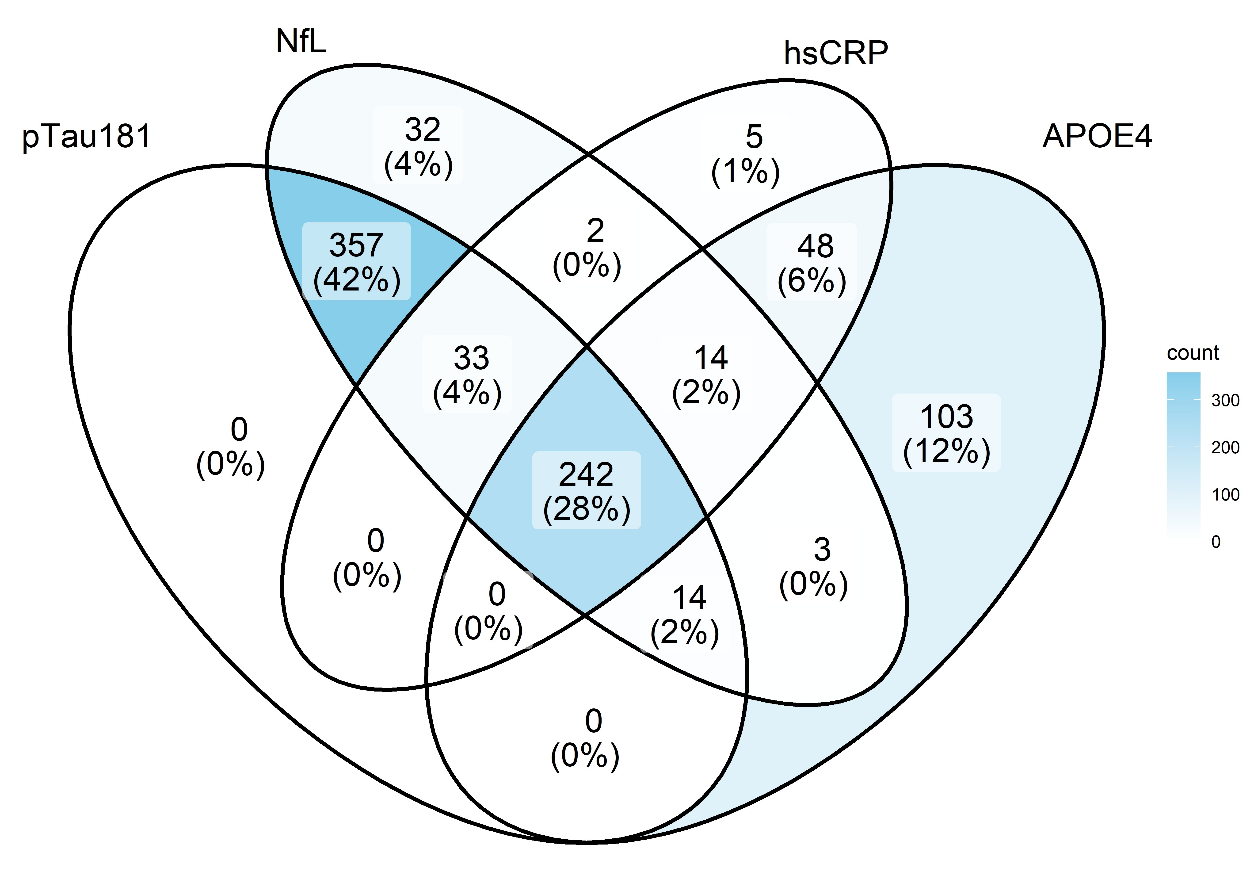


**Supplementary Figure 1. Biomarker availability overlap**. *Venn diagram displaying biomarker availability overlap between the subsamples, with the N and percentage relative to the total of participants with biomarker data (N=853) for each overlap. Abbreviations: APOE4, apolipoprotein e4; hsCRP, high-sensitivity C-reactive protein; NfL, neurofilament light; pTau181, phosphorylated tau 181.*

*
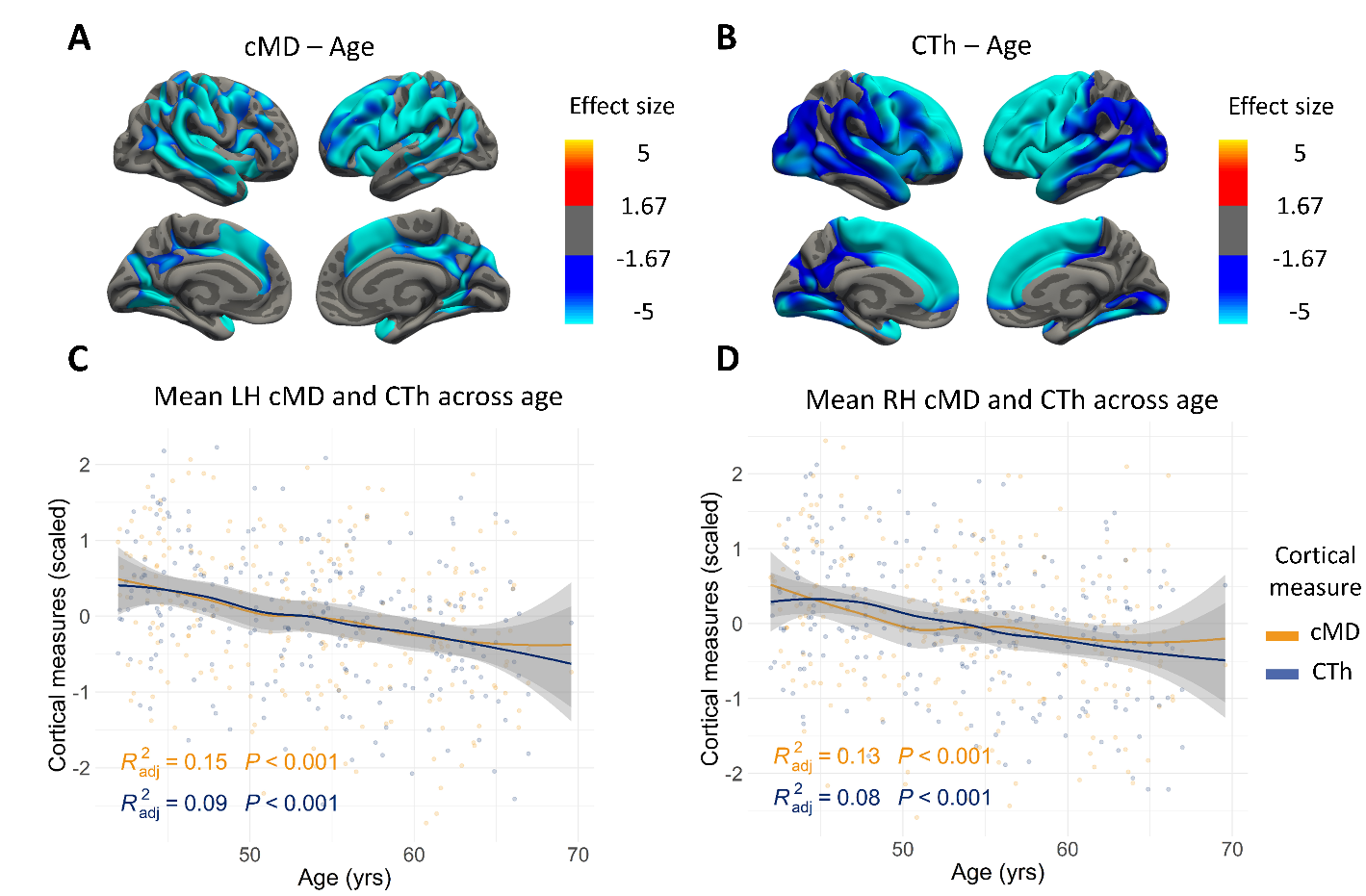
*

**Supplementary Figure 2. Associations between cMD or CTh and age in a subsample with all available biomarkers*;*** ***A)*** *Association between cMD and Age;* ***B)*** *Association between CTh and Age;* ***C)*** *Scatter plot of the association between mean cMD and CTh with age for the LH;* ***D)*** *Scatter plot of the association between mean cMD and CTh with age for the RH. Sample size for all the available biomarkers was N=242. A whole brain vertex-wise GLM was performed with brain measures (cMD or CTh) as the outcome variable and age as the predictor for A) and B). Only clusters that maintained a p-value < 0.05 after FWE multiple comparison correction are shown. Coloured thresholds were adjusted regarding the effect sizes (gamma values are scaled x10^-7­^ for cMD and x10^-2­ ­^for CTh) of the clusters for correct visualization. Scatter plots in C) and D) show the association of each metric (cMD in orange; CTh in blue) with age for the left and right hemispheres, respectively. For each cortical measure, the R-squared and p-value of the linear model covarying for sex are displayed, while the global trend is represented visually by LOESS method. In all cases, analyses are corrected for sex. Abbreviations: cMD, cortical mean diffusivity; CTh, Cortical thickness; LH, left hemisphere; LOESS, locally estimated scatterplot smoothing; FWE, family-wise error; P, p-value; R^2^-adj, R^2^-adjusted; RH, right hemisphere; yrs, years.*

**Associations between cortical metrics and age by age groups**


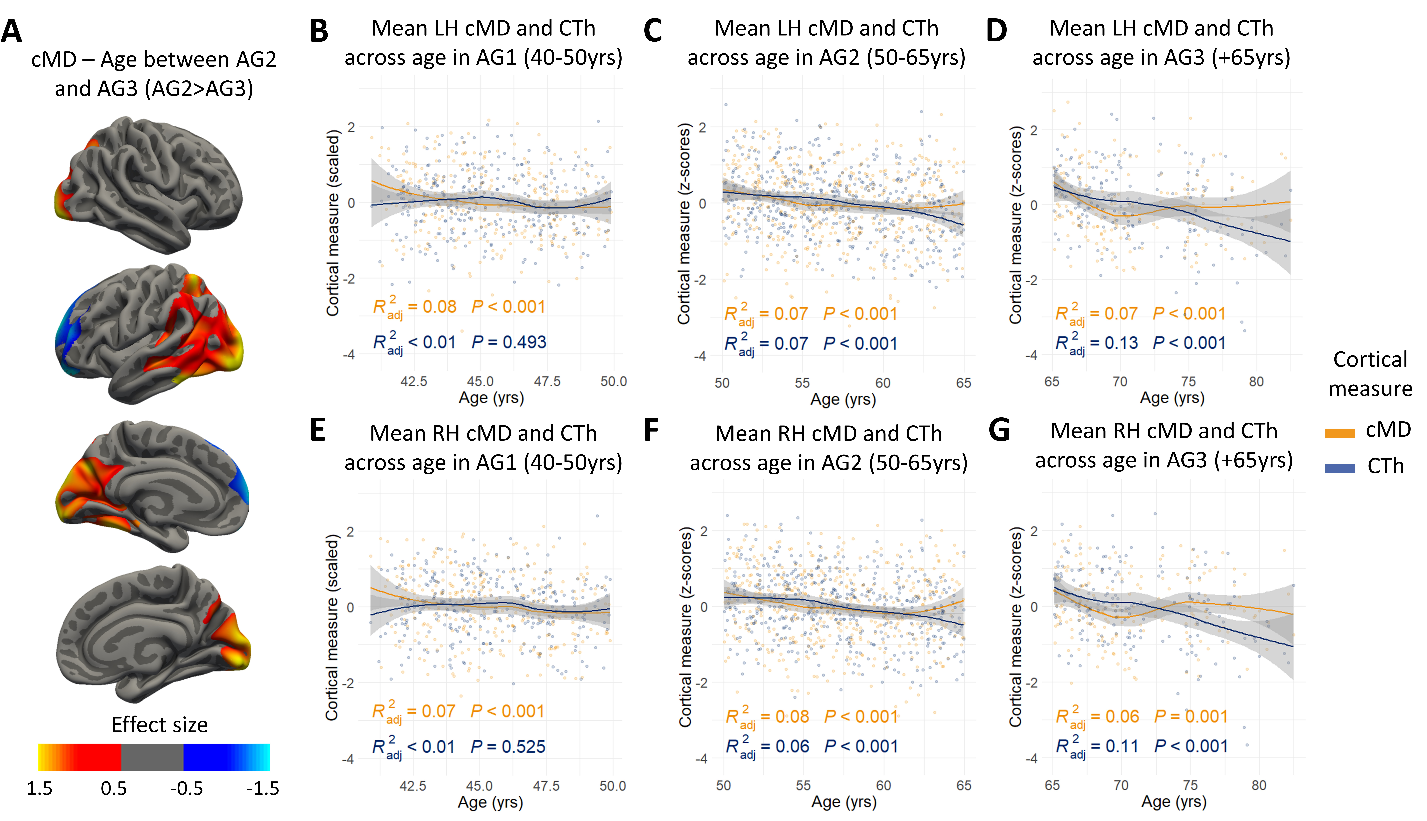


**Supplementary Figure 3. cMD or CTh associations with age for different age groups*;*** ***A)*** *Analyses comparing cMD-Age association between age group 2 and age group 3;* ***B), C), D)*** *Scatter plots show the association of each metric (cMD in orange; CTh in blue) with age for the RH in age groups 1, 2 and 3, respectively;* ***E), F), G)*** *Scatter plots show the association of each metric (cMD in orange; CTh in blue) with age for the RH in age groups 1, 2 and 3, respectively. A whole brain vertex-wise GLM was performed to test whether the age effect on cMD differed between AG2 and AG3 (contrast: AG2 > AG3) in A) (N=624).* *Only clusters that maintained a p-value > 0.05 after FWE multiple comparison correction are shown. Coloured thresholds were adjusted regarding the effect sizes (gamma values are scaled x10^-7­^) of the clusters for correct visualization. In scatter plots (B-G), cortical measure values were scaled so that they were comparable between metrics. For each cortical measure, the R-squared and p-value of the lineal model covarying for sex are displayed, while the global trend is represented visually by local polynomial regression (loess method). Age group subsamples were defined as age group 1 including volunteers aged 40 to 50 (N=300), age group 2 including volunteers from 50 to 65 years old (N=484), and age group 3 including volunteers older than 65 years (N=180). Abbreviations: AG1, age group 1; AG2, age group 2; AG3, age group 3; cMD, cortical mean diffusivity; CTh, cortical thickness; LH, left hemisphere; LOESS, locally estimated scatterplot smoothing; FWE, family-wise error; P, p-value; R^2^-adj, R^2^-adjusted; RH, right hemisphere; yrs, years.*

**Associations between cortical metrics and cognition by age groups**


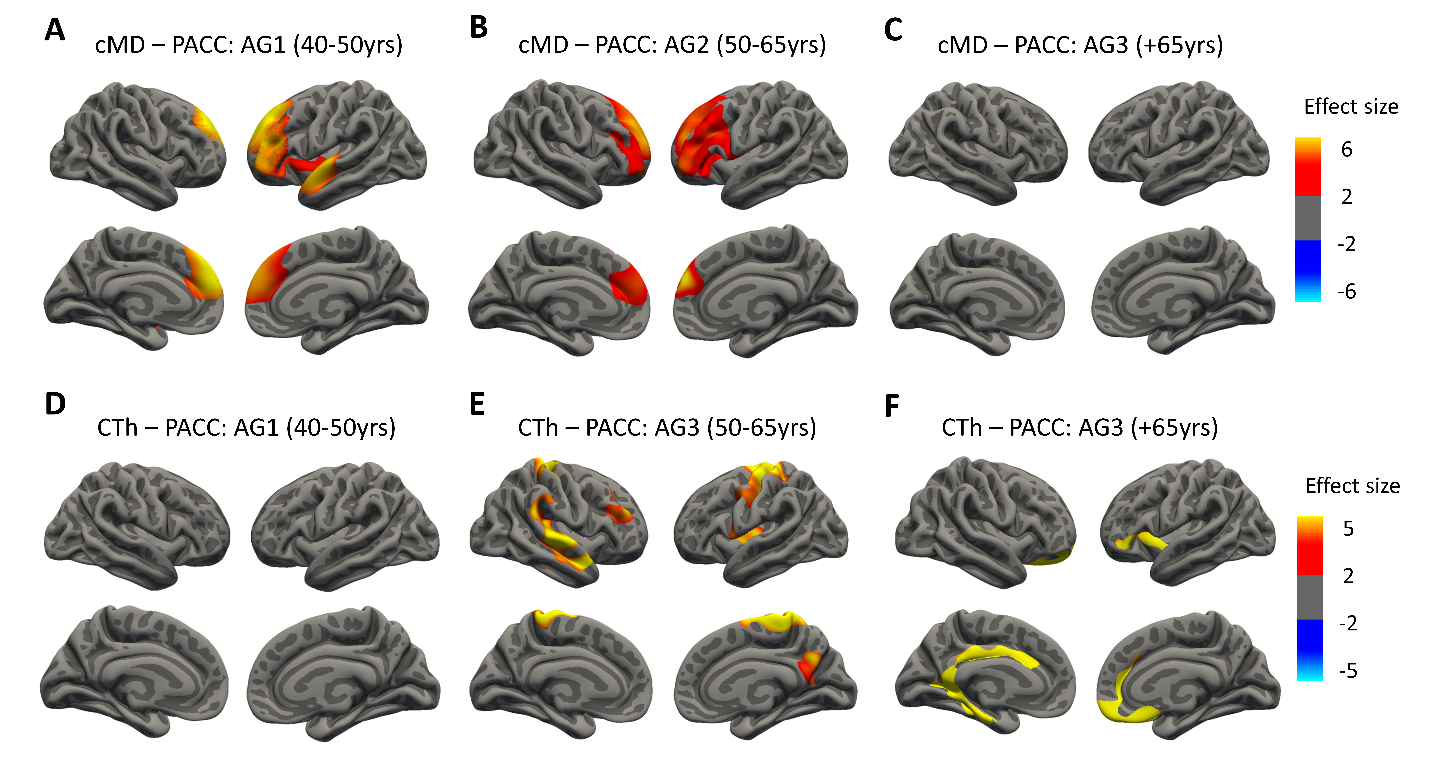


**Supplementary Figure 4. Vertex-wise whole brain associations between cMD or CTh and PACC scores by age group*;*** ***A), B), C)*** *Associations between cMD and PACC scores for age groups 1, 2 and 3, respectively;* ***D), E), F)*** *Associations between CTh and PACC scores for age groups 1, 2 and 3, respectively. A whole brain vertex-wise GLM was performed with brain measures (cMD or CTh) as the outcome variable and PACC scores as the predictor. Only clusters that maintained a p-value > 0.05 after FWE multiple comparison correction are shown. Coloured thresholds were adjusted regarding the effect sizes (gamma values are scaled x10^-7­^ for cMD and x10^-2­ ­^for CTh) of the clusters for correct visualization. Age group subsamples were defined as age group 1 including volunteers aged 40 to 50 (N=300), age group 2 including volunteers from 50 to 65 years old (N=484), and age group 3 including volunteers older than 65 years (N=180). Abbreviations: AG1, age group 1; AG2, age group 2; AG3, age group 3; cMD, cortical mean diffusivity; CTh, cortical thickness; FWE, family-wise error; PACC, Preclinical Alzheimer Cognitive Composite; yrs, years.*

**cMD levels comparison between risk profiles**


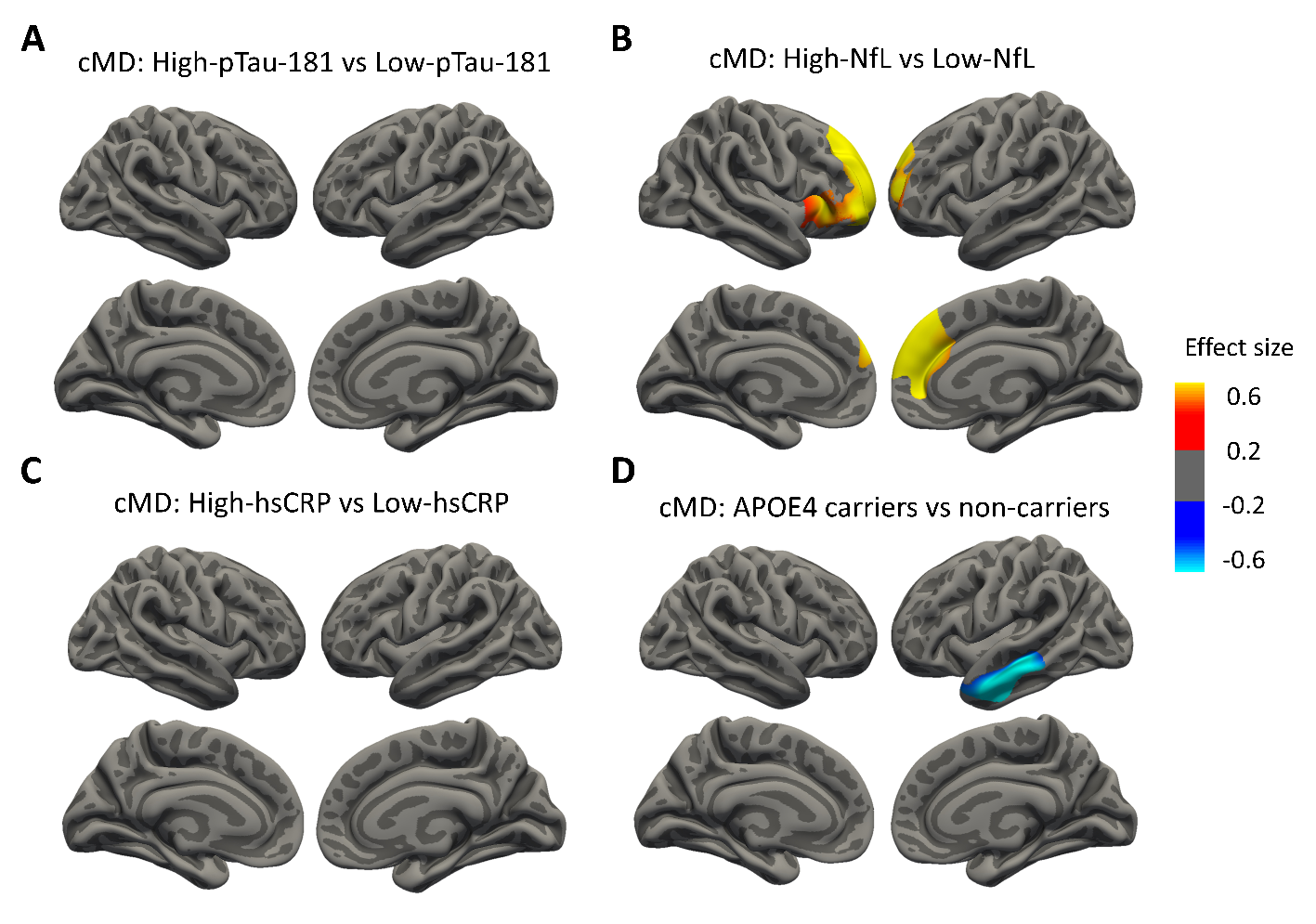


**Supplementary Figure 5. Whole-brain vertex-wise contrasts comparing cMD values between risk and non-risk profiles; A)** *cMD values in high- vs low-level pTau-181 groups (N=646);* ***B)*** *cMD values in high- vs low-level NfL groups (N=967);* ***C)*** *cMD values in high- vs low-level hsCRP groups (N=344);* ***D)*** *cMD values in APOE4 carriers vs non-carriers (N=424). A whole brain vertex-wise GLM was performed to test whether the cMD levels differed between high and low plasma biomarker levels (contrasts: High > Low) and APOE4 carriers and non-carriers (contrast: carriers>non-carriers). Only clusters that maintained a p-value > 0.05 after FWE multiple comparison correction are shown. Coloured thresholds were adjusted regarding the effect sizes (gamma values are scaled x10^-7­^) of the clusters for correct visualization. Abbreviations: APOE4, apolipoprotein e4; cMD, cortical mean diffusivity; FWE, family-wise error; hsCRP; high-sensitivity C-reactive protein; NfL, neurofilament light; pTau181, phosphorylated tau 181.*

**APOE4 non-carriers: associations between cMD and hsCRP**


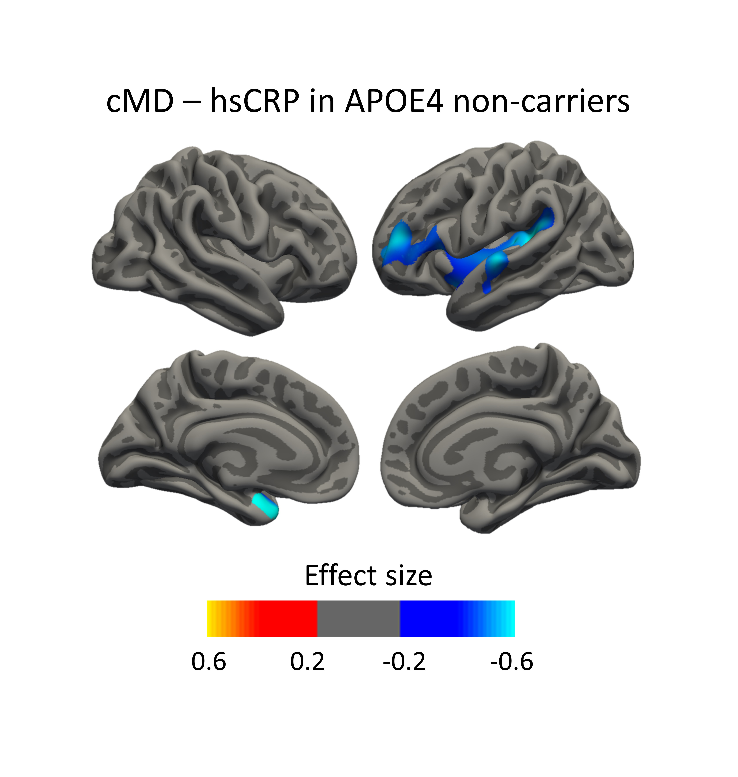


**Supplementary Figure 6. Whole-brain vertex-wise association between cMD and hsCRP plasma concentrations only for APOE4 non-carriers.** *A whole brain vertex-wise GLM was performed with cMD as the outcome variable and hsCRP levels as the predictor in a subsample of APOE4 non-carriers (N=338). Only clusters that maintained a p-value > 0.05 after FWE multiple comparison correction are shown. Coloured thresholds were adjusted regarding the effect sizes (gamma values are scaled x10^-7­^) of the clusters for correct visualization. Abbreviations: APOE4, apolipoprotein e4; cMD, cortical mean diffusivity; FWE, family-wise error; hsCRP; high-sensitivity C-reactive protein.*

**Associations between cortical metrics and age by ROI and risk profiles**


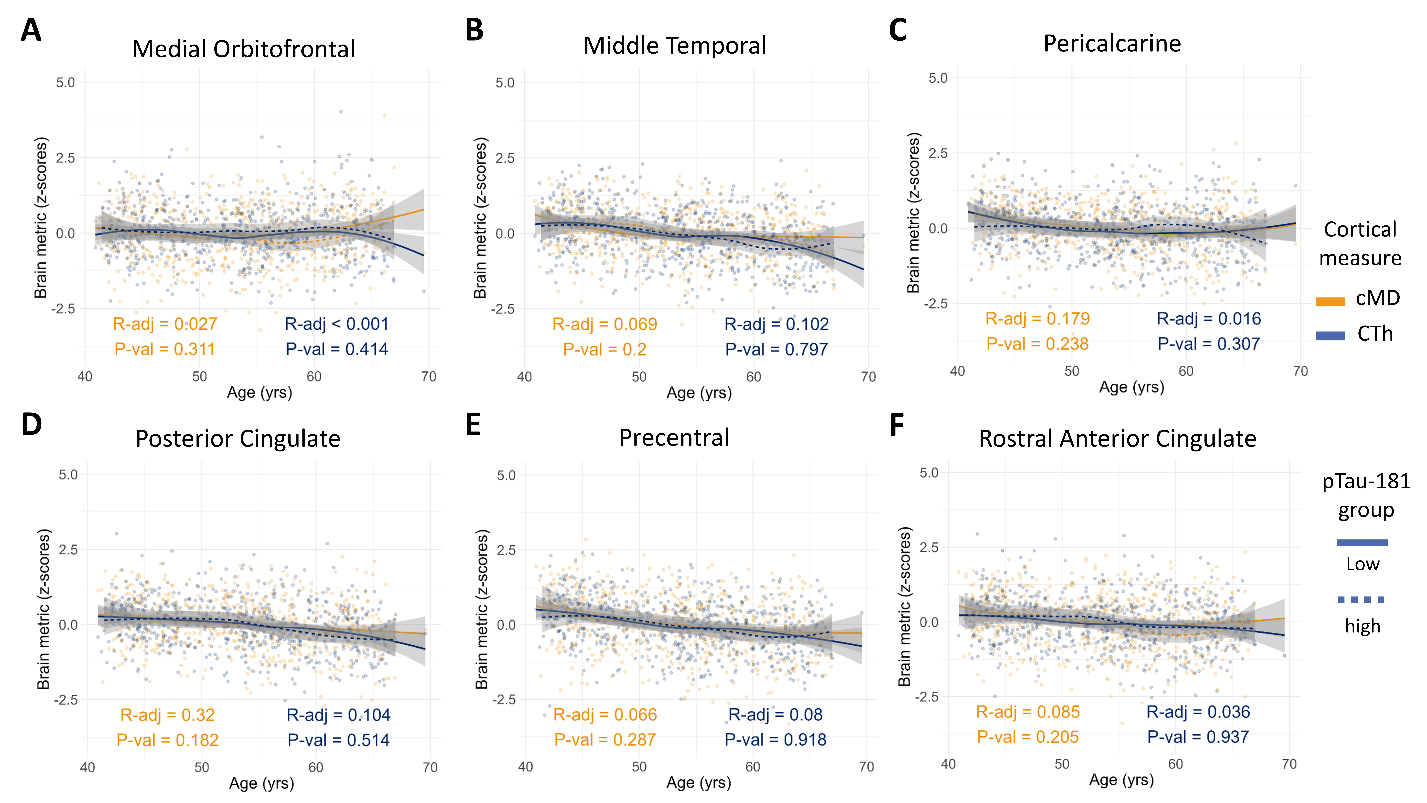


**Supplementary Figure 7.** **Scatter plots showing the association of cMD and CTh with age between high- and low-level pTau-181 for selected ROIs.** *Cortical metrics are colour-labelled (cMD in orange; CTh in blue) and high- and low-level pTau-181 groups are labelled by line type (dashed and solid, respectively). Selected ROIs are medial orbitofrontal* ***(A)****, middle temporal* ***(B)****, pericalcarine* ***(C)****, posterior cingulate* ***(D)****, precentral* ***(E)*** *and rostral anterior cingulate* ***(F)*** *cortices, and were derived from previous analyses (Figure 4). Cortical measure values were averaged between hemispheres and scaled so that they were comparable between metrics. For each cortical measure, the R-squared of the lineal model covarying for sex and including the group interaction term are displayed, while the global trend is represented visually by local polynomial regression (loess method). The slope and p-values correspond to the group interaction term in the lineal model. Sample size was that of the available biomarker data (N=646). Abbreviations: cMD, cortical mean diffusivity; CTh, cortical thickness; LOESS, locally estimated scatterplot smoothing; pTau181, phosphorylated tau 181; P-val, p-value; R-adj, R^2^-adjusted; ROI, region of interest; yrs, years.*


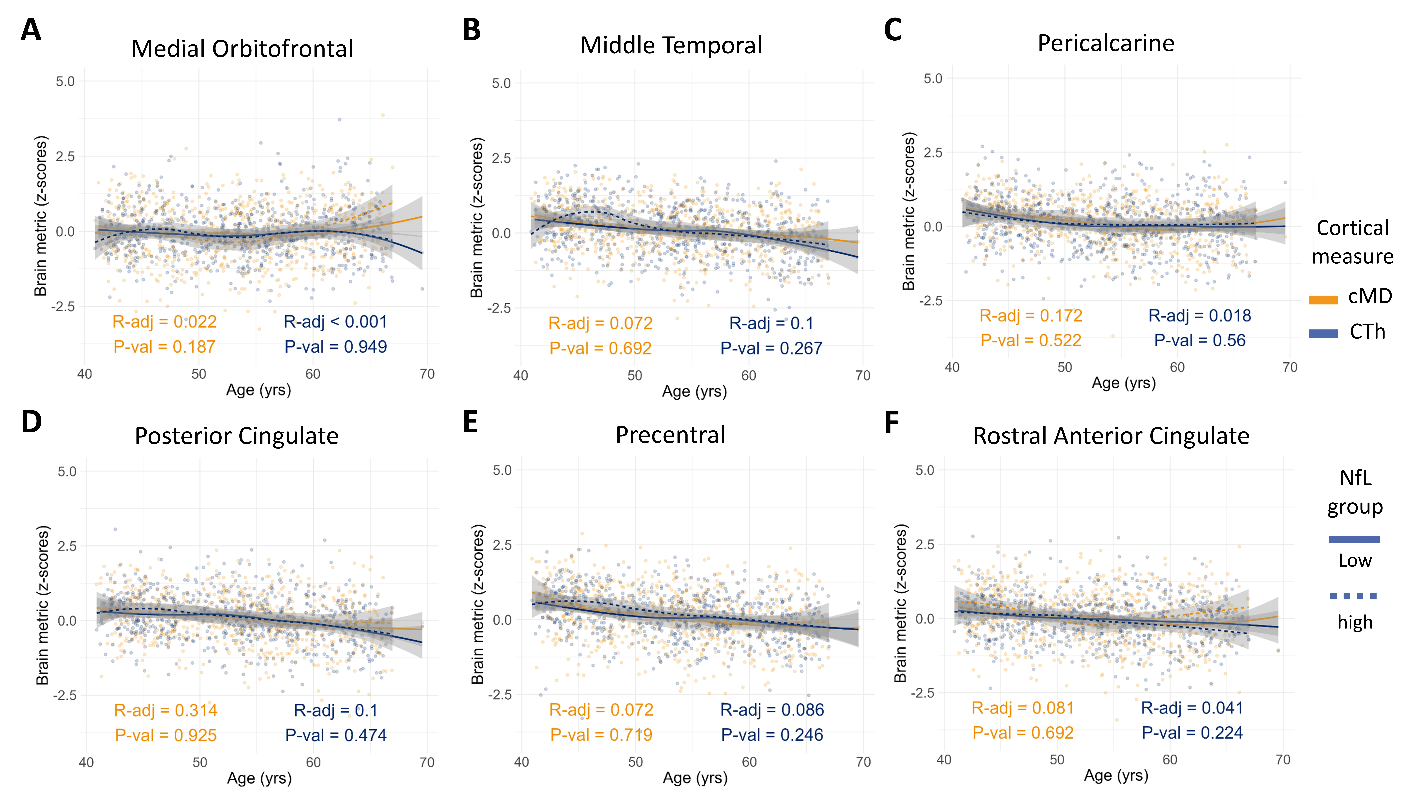
**Supplementary Figure 8.** **Scatter plots showing the association of cMD and CTh with age between high- and low-level NfL for selected ROIs.** *Cortical metrics are colour-labelled (cMD in orange; CTh in blue) and high- and low-level NfL groups are labelled by line type (dashed and solid, respectively). Selected ROIs are medial orbitofrontal* ***(A)****, middle temporal* ***(B)****, pericalcarine* ***(C)****, posterior cingulate* ***(D)****, precentral* ***(E)*** *and rostral anterior cingulate* ***(F)*** *cortices, and were derived from previous analyses (Figure 4). Cortical measure values were averaged between hemispheres and scaled so that they were comparable between metrics. For each cortical measure, the R-squared of the lineal model covarying for sex and including the group interaction term are displayed, while the global trend is represented visually by local polynomial regression (loess method). The slope and p-values correspond to the group interaction term in the lineal model. Sample size was that of the available biomarker data (N=697). Abbreviations: cMD, cortical mean diffusivity; CTh, cortical thickness; NfL, neurofilament light; LOESS, locally estimated scatterplot smoothing; P-val, p-value; R-adj, R^2^-adjusted; ROI, region of interest; yrs, years.*


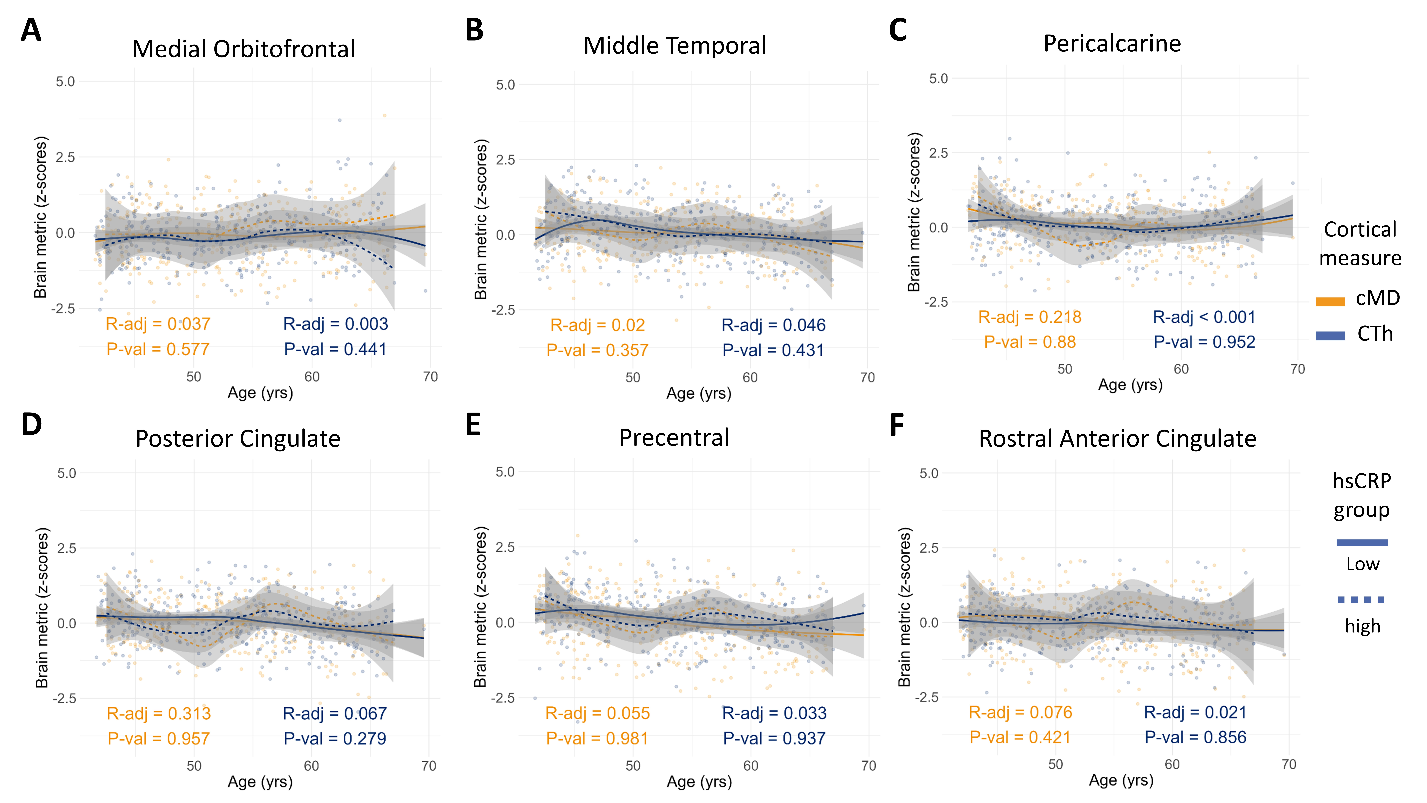
**Supplementary Figure 9. Scatter plots showing the association of cMD and CTh with age between high- and low-level hsCRP for selected ROIs.** *Cortical metrics are colour-labelled (cMD in orange; CTh in blue) and high- and low-level hsCRP groups are labelled by line type (dashed and solid, respectively). Selected ROIs are medial orbitofrontal* ***(A)****, middle temporal* ***(B)****, pericalcarine* ***(C)****, posterior cingulate* ***(D)****, precentral* ***(E)*** *and rostral anterior cingulate* ***(F)*** *cortices, and were derived from previous analyses (Figure 4). Cortical measure values were averaged between hemispheres and scaled so that they were comparable between metrics. For each cortical measure, the R-squared of the lineal model covarying for sex and including the group interaction term are displayed, while the global trend is represented visually by local polynomial regression (loess method). The slope and p-values correspond to the group interaction term in the lineal model. Sample size was that of the available biomarker data (N=344). Abbreviations: cMD, cortical mean diffusivity; CTh, cortical thickness; hsCRP, high-sensitivity C-reactive protein; LOESS, locally estimated scatterplot smoothing; P-val, p-value; R-adj, R^2^-adjusted; ROI, region of interest; yrs, years.*


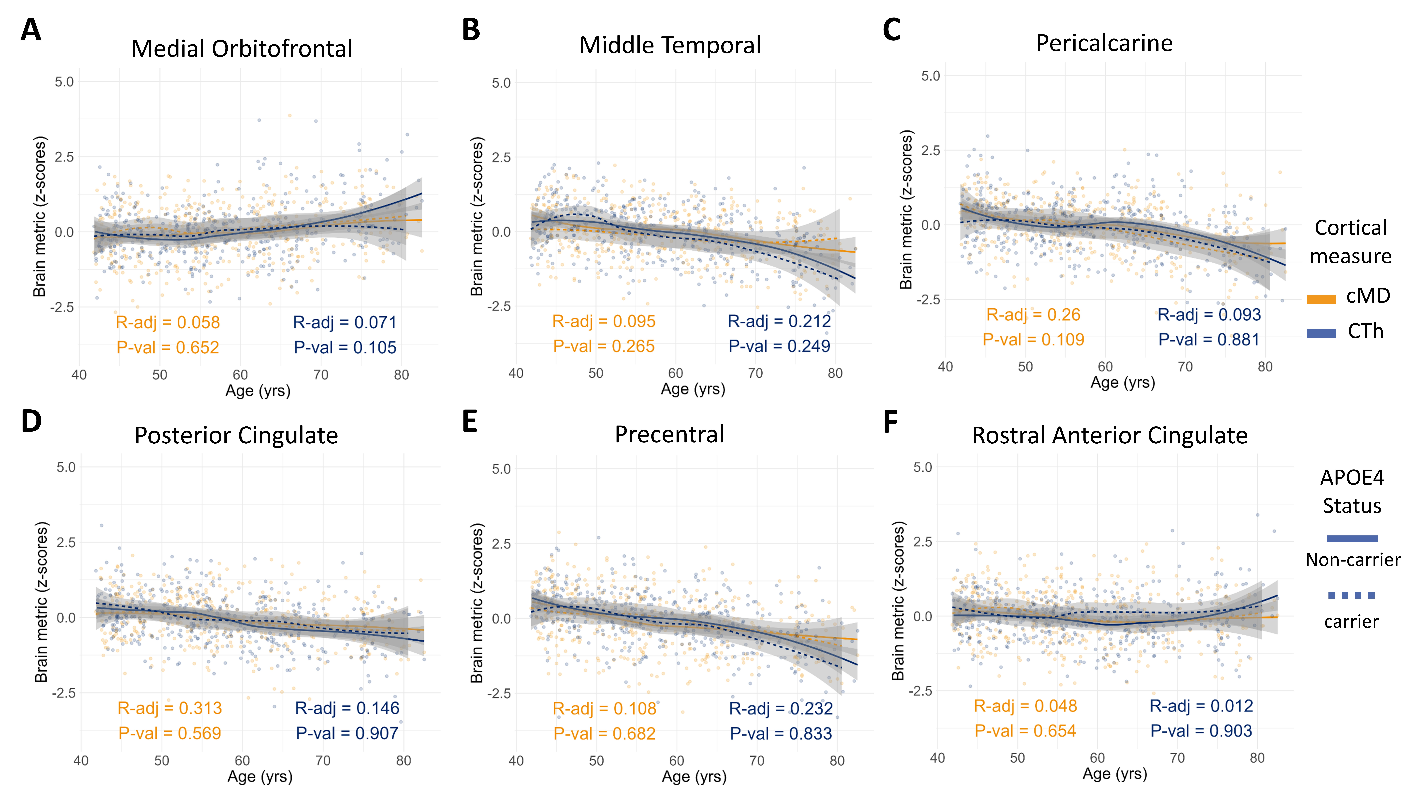
**Supplementary Figure 10. Scatter plots showing the association of cMD and CTh with age between *APOE4* carriers and non-carriers for selected ROIs.** *Cortical metrics are colour-labelled (cMD in orange; CTh in blue) and APOE4 carriers and non-carriers are labelled by line type (dashed and solid, respectively). Selected ROIs are medial orbitofrontal* ***(A)****, middle temporal* ***(B)****, pericalcarine* ***(C)****, posterior cingulate* ***(D)****, precentral* ***(E)*** *and rostral anterior cingulate* ***(F)*** *cortices, and were derived from previous analyses (Figure 4). Cortical measure values were averaged between hemispheres and scaled so that they were comparable between metrics. For each cortical measure, the R-squared of the lineal model covarying for sex and including the group interaction term are displayed, while the global trend is represented visually by local polynomial regression (loess method). The slope and p-values correspond to the group interaction term in the lineal model. Sample size was that of the available biomarker data (N=424). Abbreviations: APOE4, apolipoprotein e4; cMD, cortical mean diffusivity; CTh, cortical thickness; LOESS, locally estimated scatterplot smoothing; P-val, p-value; R-adj, R^2^-adjusted; ROI, region of interest; yrs, years.*

**Associations between cortical metrics adjusting by age, sex, and biomarker levels**


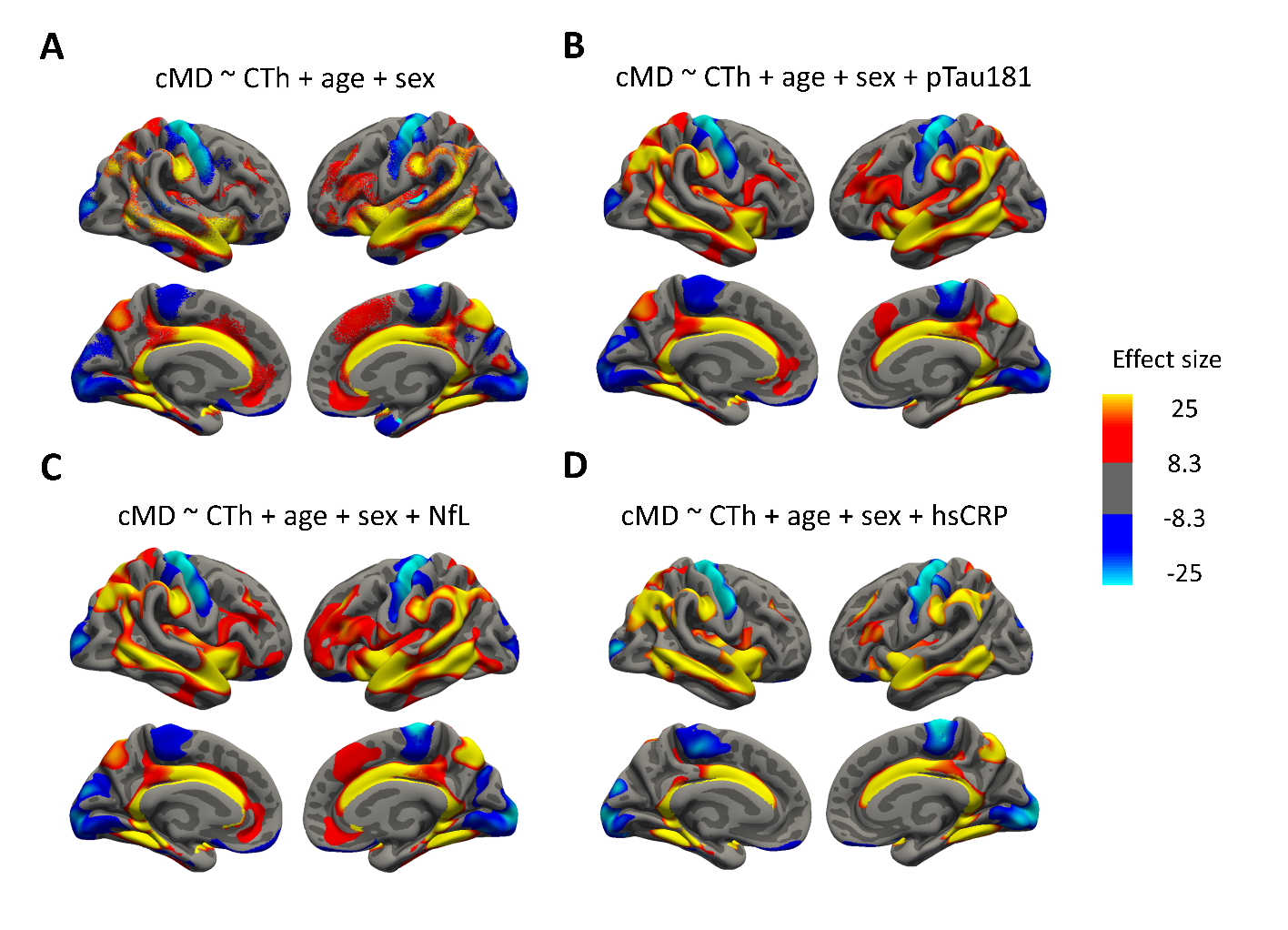
**Supplementary Figure 11. Whole-brain vertex-wise associations between cMD and CTh;** ***A)*** *Adjusted by age and sex (N=964);* ***B)*** *Adjusted by age, sex and pTau-181 levels (N=646);* ***C)*** *Adjusted by age, sex and NfL levels (N=697);* ***D)*** *Adjusted by age, sex and hsCRP levels (N=344). A whole brain vertex-wise GLM was performed with cMD as the outcome variable and CTh as the predictor, including age, sex, and plasma biomarker levels (pTau-181, NfL and hsCRP) as covariates. Only clusters that maintained a p-value > 0.05 after FWE) multiple comparison correction are shown. Coloured thresholds were adjusted regarding the effect sizes (gamma values are scaled x10^2^) of the clusters for correct visualization. Abbreviations: cMD, cortical mean diffusivity; CTh, cortical thickness; FWE, family-wise error; hsCRP; high-sensitivity C-reactive protein; NfL, neurofilament light; pTau181, phosphorylated tau 181.*

**Associations between cortical metrics by age group**


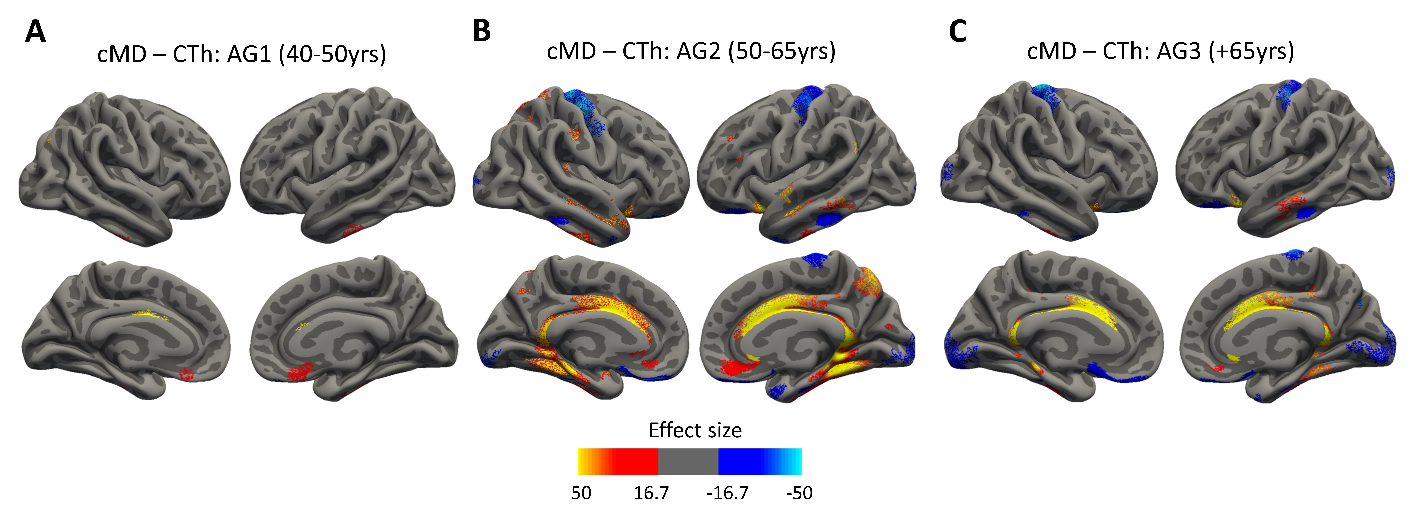


**Supplementary Figure 12. Whole-brain vertex-wise associations between cMD and CTh by age group; *A)*** *Association between cMD and CTh in age group 1.* ***B)*** *Association between cMD and CTh in age group 2 (AG2).* ***C)*** *Association between cMD and CTh in age group 3. A whole brain vertex-wise GLM was performed with cMD as the outcome variable and CTh as the predictor. Only clusters that maintained a p-value > 0.05 after family-wise error (FWE) multiple comparison correction are shown. Coloured thresholds were adjusted regarding the effect sizes (gamma values are scaled x10^2^) of the clusters for correct visualization. Age group subsamples were defined as age group 1 including volunteers aged 40 to 50 (N=300), age group 2 including volunteers from 50 to 65 years old (N=480), and age group 3 including volunteers older than 65 years (N=180). Abbreviations: AG1, age group 1; AG2, age group 2; AG3, age group 3; cMD, cortical mean diffusivity; CTh, cortical thickness; FWE, family-wise error; y, years.*
